# Gas Molecule Assisted All‐Inorganic Dual‐Interface Passivation Strategy for High‐Performance Perovskite Solar Cells

**DOI:** 10.1002/advs.202404444

**Published:** 2024-07-04

**Authors:** Fancong Zeng, Lin Xu, Jiahe Xing, Yanjie Wu, Yuhong Zhang, Huan Zhang, Chencheng Hu, Biao Dong, Xue Bai, Hongwei Song

**Affiliations:** ^1^ State Key Laboratory on Integrated Optoelectronics College of Electronic Science and Engineering Jilin University 2699 Qianjin Street Changchun 130012 P. R. China; ^2^ Key Laboratory of Bionic Engineering Ministry of Education College of Biological and Agricultural Engineering Jilin University Changchun 130022 P. R. China; ^3^ School of Physics and Electronics Henan University Kaifeng 475001 P. R. China

**Keywords:** all‐inorganic treatment, dual‐interface passivation, gas‐assisted passivation, perovskite solar cells

## Abstract

The trap states at both the upper and bottom interfaces of perovskite layers significantly impact non‐radiative carrier recombination. The widely used solvent‐based passivation methods result in the disordered distribution of surface components, posing challenges for the commercial application of large‐area perovskite solar cells (PSCs). To address this issue, a novel NH_3_ gas‐assisted all‐inorganic dual‐interfaces passivation strategy is proposed. Through the gas treatment of the perovskite surface, NH_3_ molecules significantly enhanced the iodine vacancy formation energy (1.54 eV) and bonded with uncoordinated Pb^2+^ to achieve non‐destructive passivation. Meanwhile, the reduction of the film defect states is accompanied by a decrease in the work function, which promotes carrier transport between the interface. Further, a stable passivation layer is constructed to manage the bottom interfacial defects using inorganic potassium tripolyphosphate (PT), whose ─P═O group effectively mitigated the charged defects and lowered the carrier transport barriers and nucleation barriers of PVK, while the gradient distribution of K^+^ improved the crystalline quality of PVK film. Based on the dual‐interface synergistic effect, the optimal MA‐contained PSCs with an effective area of 0.1 cm^2^ achieved an efficiency of 24.51% and can maintain 90% of the initial value after aging (10−20% RH and 20 °C) for 2000 h.

## Graphical abstract

A novel inorganic molecule dual interface passivation strategy is designed by introducing non‐destructive pure NH_3_ gas and potassium tripolyphosphate at the upper and bottom interface. The optimized PSC achieved an excellent power conversion efficiency (PCE) of 24.51%, with significant FF (81.88%) and V_OC_ (1.229V). This PCE value is the highest reported efficiency for MA‐contained PSCs employing gas‐passivation so far.

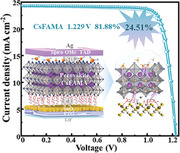

## 1 Introduction

The photovoltaic industry serves as a crucial driver for the progress and application of clean energy. Within this realm, perovskite (PVK) solar cells (PSCs) are positioned as an especially promising technological advancement, attracting ample attention for their exceptional semiconductor performance paired with their affordability.^[^ ^1^, ^2^ ^]^ Remarkably, the power conversion efficiency (PCE) of single‐junction PSCs has surged to an impressive 26.21% within a notably short period of time.^[^ ^3^ ^]^ Nevertheless, there is still a considerable gap to the theoretical Shockley‐Queisser (S‐Q) limit, which is closely associated with the significant number of inherent defects at the interfaces.^[^ ^4^, ^5^, ^6^ ^]^ Earlier studies have revealed that the high number of organic cation vacancies, interstitial ions, and excessive disharmonious Pb^2+^ defects on both the upper and bottom surfaces mainly stem from the ionic properties of PVK and the evaporation of organic components during the annealing process.^[^ ^7^, ^8^, ^9^ ^]^ Besides, not limited to the PVK, the surface of SnO_2_ at the bottom interface also shows multiple oxygen vacancies (O_V_), linked hydroxyl groups (─OH), and unsaturated coordination metal ions,^[^ ^10^ ^]^ which not only furnishe active sites for non‐radiative recombination (NRR) of charge carriers but also constitutes entry points for water and oxygen, eventually triggering degradation of device performance.^[^ ^11^, ^12^, ^13^ ^]^ Therefore, the passivation of the top surface of PVK and the bottom interface is crucial for constructing high‐performance PSCs.

There are many tailored interface strategies that have been devised to mitigate the loss of device performance triggered by interface defects. A widely used strategy for PVK surface treatment is the introduction of passivation by solution method to construct a modified layer.^[^ ^14^ ^]^ In this regard, the principal approach of passivating bottom interfaces involves incorporating polymers,^[^ ^15^ ^]^ organic small molecules,^[^ ^16^ ^]^ organic halide salts,^[^ ^17^ ^]^ or 2D materials^[^ ^18^ ^]^ as a modifying layer to refine the SnO_2_ substrates prior to the deposition of PVK precursor solutions. Nonetheless, there are practical challenges arise due to the high solubility of dimethylformamide (DMF) and dimethyl sulfoxide (DMSO). The passivation layer on the SnO_2_ substrate may be washed away during the subsequent spin‐coating process, resulting in an uneven distribution of passivation molecules on the surface of the modification layer,^[^ ^19^ ^]^ which poses a considerable restriction on the efficiency of solution‐based passivation strategies. Compared to SnO_2_, passivating the PVK surface using the solution method is more challenging. The soft ionic nature of solution‐processed PVK inevitably leads to the formation of numerous defects in the PVK films, such as uncoordinated Pb^2+^, cationic vacancies, iodine vacancies, interstitial iodine, and Pb−I antisite defects. These detrimental defects render the PVK highly sensitive to environmental factors like moisture, temperature, and light, making it prone to decomposition under adverse conditions.^[^ ^20^ ^]^ Furthermore, the use of solvents on the PVK surface can disrupt the distribution of surface components, resulting in uncertainty about the type and number of defects, and thus unsatisfactory reproducibility of manufactured devices.^[^ ^21^ ^]^ Hence, it is imperative to explore more effective treatment strategies to achieve efficient surface passivation.

Compared with the liquid‐phase and solid‐phase surface treatment, the gas‐phase surface treatment offers notable advantages, including dimensional compatibility, non‐destructive contact, and minimal residual surface passivator.^[^ ^22^ ^]^ For example, to target the passivation of uncoordinated Sn^4+^ in the PVK film, Zhang et al. utilized an ethane‐1,2‐diamine (EDA) vapor passivation method via side heating evaporation. This approach resulted in a higher short‐circuit current (J_SC_) of 24.05 mA·cm^−2^.^[^ ^23^ ^]^ Wang et al. used a similar strategy through evaporating a 99% CS_2_ solution to passivate the iodine vacancies (V_I_), which effectively enhanced the long‐term stability of the FAPbI_3_ devices, maintaining more than 90% of the initial efficiency after 2000 h (30% RH, 30 °C).^[^ ^22^ ^]^ These works fully proved the effectivity of the gas‐phase non‐destructive surface passivation strategy. However, the gas‐phase passivators employed in the current research all rely on evaporating the precursor solutions. Such an evaporation process introduces challenges such as uncontrollable evaporation rates, lower vapor purity as well as vapor escape. This hinders the achievement of controlled variables and reduces the consistency and reliability of gas‐phase passivation. Therefore, there is a pressing need to develop a more precise and controllable method for gas‐phase passivation to further enhance its effectiveness.

**Scheme 1:**
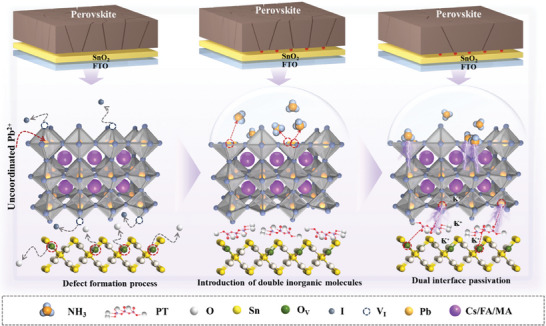
The diagram of NH_3_ assisted the all‐inorganic dual‐interface passivation strategy.

Herein, we proposed an all‐inorganic dual‐interface passivation strategy dominated by pure inorganic gas of NH_3_, as shown in **Scheme** **1**. An efficient and non‐destructive top surface passivation of PVK was employed by precise injection of different concentrations of NH_3_ under a nitrogen atmosphere and high temperature. The passivation of uncoordinated Pb^2+^ and V_I_ on the surface of PVK films was achieved through in‐situ pure NH_3_ gas passivation. Due to the effective management of the surface defects of PVK, the corresponding work function (*W_F_*) was optimized and the residual stress (ɛ) was also alleviated, which was beneficial for improving the carrier transfer rate and the stability of PVK. In addition, due to the inherently stable structural characteristics of SnO_2_, it is not easy to interact directly with gases. Therefore, potassium tripolyphosphate (PT), an inorganic small molecule insoluble in organic solvents like DMSO and DMF, was introduced at the bottom interface to further achieve all‐inorganic dual‐interface passivation. Leveraging the negative charge group ─P═O in PT, we aimed to suppress specific defects at the SnO_2_‐PVK interface, thereby promoting carrier transport. Furthermore, the crystallization of PVK was expected to be improved by utilizing the vertical gradient distribution of K^+^ contained in PT. Based on the above synergistic effects, the PSC of 0.1 cm^2^ showed an exciting PCE of 24.51%. And, even after 2000 h of exposure to air, the unpackaged device could maintain 90% of its initial efficiency.

## 2 Results and Discussion

**Figure 1:**
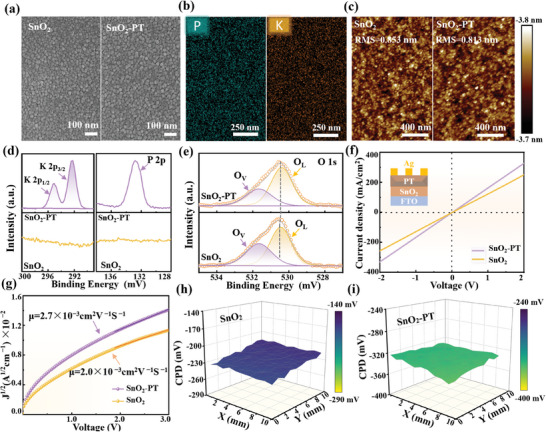
The SEM images of a) SnO_2_ and SnO_2_‐PT films. EDS mapping images of b) K and P elements in SnO_2_‐PT thin film. The AFM images of c) SnO_2_ and SnO_2_‐PT films. High‐resolution XPS spectra of SnO_2_ and SnO_2_‐PT films: d) P 2p, K 2p, and e) O 1s. f) The I‐V characteristics of FTO/SnO_2_/Ag and FTO/SnO_2_‐PT/Ag devices. g) The carrier mobility of SnO_2_ and SnO_2_‐PT films determined by SCLC test. CPD of h) SnO_2_ and i) SnO_2_‐PT films.

To establish an efficacious gas‐assisted all‐inorganic dual‐interface passivation strategy, first, the bottom interface between SnO_2_ and PVK was optimized by introducing inorganic small molecule PT since the semiconductor oxide is stable and largely unaffected under gas treatment. The structure of the PT molecule was exhibited in Figure S1a (Supporting Information), composed of K^+^ and triphosphate groups. Figure S1b (Supporting Information) depicts the electrostatic surface potential (ESP) plot of PT calculated from density functional theory (DFT), where the triphosphate group (three ─P═O groups) exhibit higher electron density (such as negatively charged groups, red), while the K^+^ group exhibits lower electron density (such as positively charged groups, blue). **Figure** **1**a displays the top‐view scanning electron microscope (SEM) images of SnO_2_ thin films before and after PT modification. There was no significant change in the grain size of SnO_2_ thin film after PT surface treatment (Figures S2 and S3, Supporting Information). The average size of the grains of all the films was distributed in the range of 21–23 nm (Table S1, Supporting Information). The thickness of the SnO_2_ films before and after the PT surface treatment was measured by a stylus profiler (Figures S4–S7, Supporting Information). As expected, the PT molecules might be distributed on the SnO_2_ grains without forming a continuous thin layer, thus not causing a significant change in the thickness of the SnO_2_ films. Energy dispersion spectrum (EDS) mapping and total spectrum analysis were conducted to assess the PT distribution on the SnO_2_ surface (Figure 1b; Figure S2, Supporting Information). The EDS mapping shows a uniform distribution of elements like oxygen (O), tin (Sn), phosphorus (P), and potassium (K). Meanwhile, the prevalence of P and K peaks in the total spectrum substantiates the presence of PT on the SnO_2_ surface. In addition, the illustrations in Figure S8 (Supporting Information) show the elemental content ratios of Sn, O, P, and K, which also indicates the presence of PT. The atomic force microscopy (AFM) tests were carried out to further demonstrate the beneficial impact of PT introduction on the morphology of SnO_2_ film (Figure 1c). It enabled a smoother surface, a finding consistent with SEM images.

X‐ray photoelectron spectroscopy (XPS) was used to analyze the surface elements and chemical states of SnO_2_ and SnO_2_‐PT films. As outlined in Figure 1d,e, the characteristic peaks of P 2p and K 2p originating from PT were detected solely in the SnO_2_‐PT film, further proving the successful introduction of PT into the SnO_2_ film. Further, the Sn 3d peak of SnO_2_‐PT shifted toward a lower binding energy by 0.14 eV, indicating an increase in electron density around the Sn atom (Figure S9, Supporting Information).^[^ ^24^ ^]^ The O 1s spectra for both films could be deconvolved into two peaks: lattice oxygen (O_L_) and O_V_.^[^ ^25^ ^]^ Compared to the pristine SnO_2_ film, the intensity of O_V_ in SnO_2_‐PT film considerably reduced, with peak area change from 41.0% to 35.0%. Alternatively, the peak area for O_L_ increases substantially from 59.0% to 65.1%. Observations from the XPS energy spectral changes in Sn 3d and O 1s highlight the interaction between PT and SnO_2_ films. Specifically, the ─P═O functional group in inorganic PT molecules can serve as a Lewis base and coordinate with the uncoordinated Sn caused by O_V_.^[^ ^26^ ^]^ This process decelerates the reduction of Sn^4+^, which ultimately results in decreased O_V_. Additionally, this interaction doesn't affect the pristine transmittance of SnO_2_ film in the visible light range (300–800 nm) even with different PT modification concentrations, as revealed in Figure S10a (Supporting Information).

The electrical performance of SnO_2_ as an electron transport layer (ETL) plays a crucial role in reducing the open‐circuit voltage (V_OC_) loss of PSCs. In order to investigate the influence of PT on the conductivity of SnO_2_ film, the current‐voltage (I‐V) curves of the device with the structure of FTO/SnO_2_ or SnO_2_‐PT/Ag were measured with different concentrations of PT modifications (Figure S10b, Supporting Information). The conductivity of the corresponding SnO_2_‐PT film was determined and recorded in Table S2 (Supporting Information). As the concentration of PT increases to 3 mg mL^−1^, the conductivity of SnO_2_‐PT transitioned from 3.75 × 10^−6^ to 4.93 × 10^−6^ S·cm^−1^ due to the reduction of the trap density of O_V_ on the surface of SnO_2_, as analyzed in the above XPS results. However, as the PT concentration continued to increase, the conductivity began to depict a decreasing trend. This phenomenon can be traced back to an excessive introduction of PT, which in turn obstructs carrier transport. According to I‐V testing, we determined that the SnO_2_‐PT films had optimal conductivity at a PT concentration of 3 mg mL^−1^ (Figure 1f). Therefore our subsequent discussions of SnO_2_‐PT films are based on the PT concentration of 3 mg mL^−1^. To determine the density of defect states and the mobility of carriers for SnO_2_ and SnO_2_‐PT films, an associated electronic sponding space charge limited current (SCLC) curve was measured, as illustrated in Figure 1g and S11 (Supporting Information). Devices with FTO/(SnO_2_ or SnO_2_‐PT)/phenyl‐C61‐butyric acid methyl ester (PCBM)/Ag structures were prepared.^[^ ^27^ ^]^ As calculated, the trap‐filled limit voltage (N_t_) value of SnO_2_‐PT film (4.11 × 10^16^ cm^−3^) is lower than that of pristine SnO_2_ film (5.73 × 10^16^ cm^−3^). While for electron mobility, SnO_2_‐PT film exhibited a higher electron mobility (2.7 × 10^−3^ cm^2^·v^−1^·s^−1^) than the pristine SnO_2_ (2.0 × 10^−3^ cm^2^·v^−1^·s^−1^). This conclusion is attributed to the decrease in defect state density effectively alleviates the carrier scattering and trapping in SnO_2_ crystals, thereby improving the electrical performance of SnO_2_.

The characteristics of electronic energy band gap (E_g_) structures of SnO_2_ and SnO_2_‐PT were delineated by UV photoelectron spectroscopy (UPS) (Figure S12, Supporting Information). Taking into account of the secondary electron cutoff (hν = 21.22 eV), the calculated Fermi level (*E_f_*) of SnO_2_ film shifted directionally from −4.81 to −4.64 eV after the modification of PT, implying a decrease in *W_F_*. Following this, the surface potential difference (CPD) was gauged using a Kelvin Probe Force Microscope (KPFM). Compared the curved surface distribution in Figure 1h,i, the surface of SnO_2_‐PT brandished a much lower average surface potential (−332 mV) in contrast with that of SnO_2_ (−233 mV), it revealed that PT treatment could effectively reduce the CPD ($CPD\;=\frac{W_{tip}-W_{sample}}{-e}\;$) value, where W_tip_ and W_sample_ represent the *W_F_* of the tip and the sample, respectively.^[^ ^28^ ^]^ The reduction of CPD value directly confirms a decrease in *W_F_*, which is consistent with the results in Figure S12 (Supporting Information). Based on the above‐mentioned decrease in *W_F_* and upward shift in *E_f_*, the electron concentration in the SnO_2_ conduction band maximum (CBM) would be increased, thus increasing the conductivity of SnO_2_. This is in agreement with the measured SnO_2_‐PT conductivity results.^[^ ^29^ ^]^

**Figure 2:**
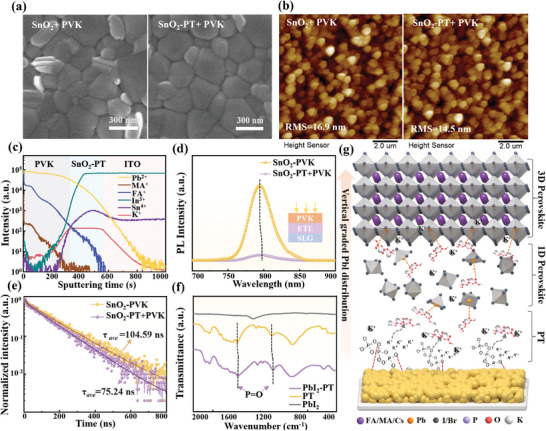
a) SEM and b) AFM images of PVK thin films deposited on SnO_2_ and SnO_2_‐PT ETLs. c) ToF‐SIMS depth profiles of Pb^2+^, MA^+^, FA^+^, In^3+^, Sn^4+^ and K^+^ of FTO/SnO_2_‐PT/PVK. d) PL and e) TRPL spectra of PVK films on SnO_2_ and SnO_2_‐PT ETLs. f) FTIR spectra of PbI_2_, PbI_2_‐PT, and PT powder. g) Schematic diagram of PT modification mechanism at the bottom interface.

Subsequently, the PVK were deposited on SnO_2_ and SnO_2_‐PT films respectively and corresponding SEM images were exhibited in **Figure** **2**a. An inspection of the SEM images reveals noticeable experiential differences in film texturing. PVK film on the SnO_2_‐PT substrate are evidently smoother and flatter, knit tighter by an enlargement in grain size. This conclusion drawn from the above SEM analysis gained additional confirmation from the AFM testing. As demonstrated in Figure 2b, the roughness of PVK on SnO_2_‐PT film diminishes from 16.9 to 14.5 nm in comparison to the PVK deposited on the pristine SnO_2_. To further ferret out the mechanisms contributing to this notable improvement in PVK crystal quality, assessments of hydrophobic angles were performed to evaluate the hydrophilic state of the substrate film. Through the hydrophobicity angle tests of SnO_2_ and SnO_2_‐PT substrates (Figure S13, Supporting Information), it was found that the hydrophobicity angle of the SnO_2_‐PT membrane decreased, indicating an increase in hydrophilicity. The contributing forces at play in this variety of changes can be elucidated by Gibbs' free energy equation for non‐uniform nucleation.^[^ ^30^ ^]^ The smaller contact angle presented by SnO_2_‐PT contributes to lower surface energy, thereby reducing the nucleation hurdle of PVK crystals and offering favorable conditions for the nucleation and subsequent crystallization of high‐quality PVK films.^[^ ^31^ ^]^

In addition, thanks to the distinctive solubility characteristics of PT, it is expected that it could be well retained at the interface layer even after the surface spin‐coating of PVK layer. The dissolution states of PT viewed in water, DMSO, and DMF were displayed in Figure S14 (Supporting Information). The foggy states of PT in DMSO and DMF solutions versus water suggested insolubility of PT in the DMSO and DMF. Therefore, we anticipate that PT particles anchored to the SnO_2_ surface may potentially permeate into the PVK film thereby assisting in its crystallization process. Depth‐dependent time‐of‐flight secondary ion mass spectrometry (ToF‐SIMS) test was executed as corroboration. As demonstrated in Figure 2c, the consequential distribution of PT is predominantly concentrated at the coupled SnO_2_/PVK bottom interface, with a measured portion diffusing into the lower PVK film strata. The gradient distribution of PT offered palpable benefits for passivating defects, particularly those noted at the SnO_2_ interface and within the bottom layers of PVK. Current studies have confirmed that K^+^ dispersion within PVK could facilitate its crystallization.^[^ ^32^ ^]^ Drawing from the analysis, the PT modified SnO_2_ offered not just an amiable substrate for PVK deposition, but the gradient distribution of K^+^ also lent momentum to the crystalline growth of the eventual PVK film.

To investigate the impact of the successful introduction of PT on the carrier recombination dynamics at the bottom interface, tests of steady‐state photoluminescence (PL) and time‐resolved photoluminescence (TRPL) were undertaken. As shown in Figure 2d,e, the PVK films deposited on the pristine SnO_2_ film displayed significantly higher PL intensity than that of the SnO_2_‐PT film. An obviously blue shift in the PL peak position was registered, transitioning from 793.8 to 793.1 nm. This outcome revealed not only a decline in defect density the SnO_2_‐PT film but also noteworthy restraints on NRR with an enhancement of extraction of interface carriers.^[^ ^33^ ^]^ From TRPL examinations, it is found that the carrier lifetime of PVK film deposited on the SnO_2_‐PT ETL surface was shortened to 28.1% of its pristine lifetime, indicating the improved carrier transport ability at the interface. The fitted time constants were recorded in Table S3 (Supporting Information). The analysis of TRPL test results further proved that the trap‐assisted NRR of PVK was reduced by the bottom interface treatment of PT, resulting in good interface transfer quality.

To further understand the mechanism mitigating of defective state‐assisted NRR, Fourier transform infrared spectroscopy (FTIR) was employed to probe the interaction between PT and PVK. Since a large amount of PbI_2_ inevitably remains at the bottom during the crystallization of PVK from one to 3D, it makes this interaction comes from PT with PbI_2_ to a greater extent. Therefore, we performed FTIR tests on PbI_2_, PT, as well as the mixture of PT and PbI_2_ as shown in Figure 2f. Besides the characteristic peak of the PT and PbI_2_ in the mixture of PT and PbI_2_, the tensile vibration peaks attributed to ─P═O shifted from 1096.2 and 952.3 cm^−1^ to 1095.1 and 946.6 cm^−1^, respectively.^[^ ^34^ ^]^ This lends evidence to intensive interactions between PT and PbI_2_, attributed to the transfer of lone pairs of electrons carried by ─P═O to uncoordinated Pb^2+^ to achieve Lewis acid‐base coordination. This result leads to a reduction in the defect state (uncoordinated Pb^2+^) at the bottom interface, promoting carrier transport. Among them, the uncoordinated Pb^2+^ is usually derived from the decomposition of the very poorly photosensitive PbI_2_ under low‐light conditions.

According to the above analysis, it is evident that the successful introduction of PT at the bottom interface has yielded a dual passivation effect (Figure 2g). First, concerning the SnO_2_ ETL at the bottom interface, the electron‐rich groups (three ─P═O) of PT inhibited the reduction of the undercoordinated Sn^4+^ on the surface by electrostatic interactions and suppressed the formation of O_V_, thereby enhancing its morphology and conductivity. Additionally, the *W_F_* of SnO_2_ was reduced by the surface treatment of PT to achieve its conductivity enhancement. Second, regarding the PVK at the bottom interface, crystal growth was effectively promoted by the longitudinal distribution of K^+^, while the negatively charged groups of ─P═O in PT acted as Lewis bases to effectively passivate the residual PbI_2_ on the bottom surface, thereby promoting the carrier transport. As an effective inorganic surface passivator, PT molecules successfully constructed a favorable bottom interface by simultaneously passivating.

**Figure 3:**
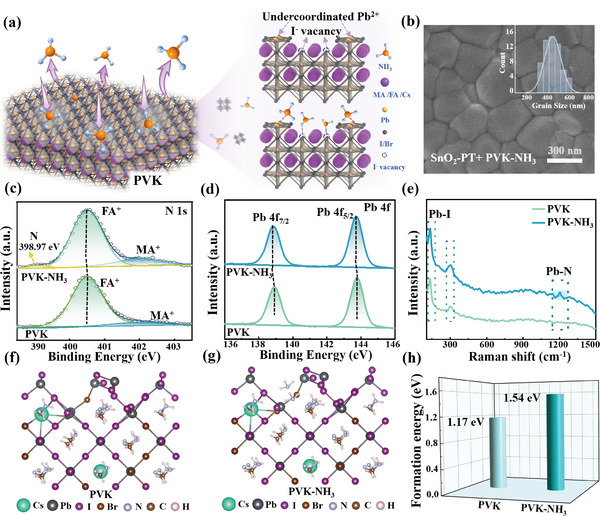
a) The schematic illustration of NH_3_ gas passivated PVK film and the NH_3_ gas passivation mechanism to PVK. b) The SEM image of PVK film deposited on SnO_2_‐PT substrate treated with NH_3_ and the corresponding particle size distribution (inset). c) The N 1s and d) Pb 4f XPS spectra of PVK and PVK‐NH_3_ film. e) Raman spectra of the PVK and PVK‐NH_3_ films. Theoretical calculation section: f,g) DFT‐based modeling of the formation energy of V_I_ on the (001) surface of PVK and h) formation energy of surface V_I_ without and with NH_3_ treatment.

Based on a good bottom interface obtained through inorganic PT molecules, the PVK surface was further treated with inorganic NH_3_ gas without solid‐liquid contact to achieve effective surface passivation of PVK. As shown in **Figure** **3**a, the obtained PVK film was annealed at 100°C for 10 min, during which NH_3_ gas with different concentrations was injected. Figure S15 (Supporting Information) shows the ESP map of NH_3_, where N atoms in NH_3_ exhibit higher electron density (such as negatively charged groups, red), while H atoms exhibit lower electron density (such as positively charged groups, blue). Therefore, during the post‐treatment process, the N atom with isolated electron pairs in NH_3_ could capture the uncoordinated Pb^2+^ on the surface of PVK. Moreover, the H atoms in NH_3_ tend to interact with the more electronegative I^−^, which was expected to inhibit I^−^ migration and passivate the iodine vacancy (V_I_). It is worth noting that during the interaction stage between NH_3_ and PVK, the defect‐free area on the PVK surface hardly interacts with NH_3_, thus contributing to a non‐destructive and targeted passivation of the PVK surface (Figure 3a).

After NH_3_ gas treatment, an increase in N element content and distribution was clearly observed in EDS mapping and total elemental distribution spectroscopy (Figure S16 and Table S4, Supporting Information), revealing the successful adsorption of NH_3_ on the PVK surface. Meanwhile, the SEM image shows that the PVK‐NH_3_ film surface is more flat and smooth (Figure 3b). This can be further confirmed in the AFM images (Figure S17, Supporting Information), in which the roughness of the PVK‐NH_3_ film was significantly reduced to 13.8 nm. When compared to the particle size distributions of other films, the PVK‐NH_3_ film displayed a relatively uniform distribution with larger‐sized particles, as depicted in Figure 3b and S18 (Supporting Information). This suggests that the introduction of NH_3_ enhanced the crystal quality of the film. The passivation effect of NH_3_ on PVK was further characterized by XPS. In the XPS energy spectrum of N 1s, the N atoms mainly originate from FA^+^ and MA^+^ cations (Figure 3c). However, a distinct N atom peak at 398.97 eV in the PVK‐NH_3_ film was observed, which was derived from NH_3_, further confirming the successful adsorption of NH_3_ on the surface of PVK (Figure 3c).^[^ ^35^ ^]^ In addition, the binding energy peaks of Pb 4f_7/2_ and Pb 4f_5/2_ located at 138.20 and 143.09 eV in PVK‐NH_3_ thin films shift toward lower binding energies obviously (Figure 3d), and a similar phenomenon can be found in I 3d spectra (Figure S19, Supporting Information). This is a manifestation of the increased electron density around Pb^2+^ on the surface of PVK‐NH_3_ film, meaning a robust interaction between Pb^2+^ ions and NH_3_.^[^ ^36^ ^]^

Raman spectroscopy was employed to probe the situation of bonding between uncoordinated Pb^2+^ on the surface of PVK and N atoms in NH_3_ (Figure 3e). The vibrational peaks were found at 133 and 300 cm^−1^, which can be assigned to Pb−I bonding in PVK.^[^ ^37^ ^]^ After the NH_3_ treatment, the Raman spectrum for the PVK‐NH_3_ film displays a small new peak at 1203 cm^−1^, which belongs to the vibrational peak of the Pb─N bond.^[^ ^37^ ^]^ This occurrence arose from the interaction between the N atom with lone electron pairs in NH_3_ and the uncoordinated Pb^2+^ in the Pb−I framework.^[^ ^38^ ^]^ Moreover, the formation of these Pb─N bonds indicates that NH_3_ adsorption is chemical adsorption. Therefore, NH_3_ not only stably adsorbs on the surface of PVK, but also achieves targeted passivation of uncoordinated Pb^2+^ and V_I_ defects on the surface of PVK. For a deeper understanding the passivation mechanism of NH_3_, DFT was conducted to evaluate the binding affinity between V_I_, I^−^, and NH_3_. Figure S20a,b (Supporting Information) depicts the adsorption states of NH_3_ with V_I_ and I^−^ of the PVK (001) crystal plane. The corresponding adsorption energies were calculated to be 1.09 and 0.57 eV, respectively (Figure S20c, Supporting Information), the binding energy between NH_3_ and V_I_ is almost twofold higher than that of NH_3_ and I^−^. This result indicates that the interaction between V_I_ from PVK and NH_3_ is more favorable than I^−^, allowing for stronger coordination with uncoordinated Pb^2+^ defect sites in the Pb−I framework. Therefore, NH_3_ molecule prefer to occupy V_I_, thereby enhancing the formation energy of V_I_. To confirm this conclusion, the formation energy of V_I_ based on the (100) crystal plane of PVK was also evaluated, as shown in Figure 3f,g. The formation energy of V_I_ after NH_3_ gas treatment (1.54 eV) is significantly higher than the untreated one (1.17 eV), implying that NH_3_ gas targeted passivation effectively increased the threshold of forming shallow defect V_I_ in I^−^ migration (Figure 3h). Thus, the NH_3_ gas passivation strategy serves not only to mitigate surface defects but also to allow robust coordination with Pb^2+^, consequently enhancing the stability of the PVK's octahedral framework. Such improvements facilitate enhancing the interfacial performance of devices.

**Figure 4:**
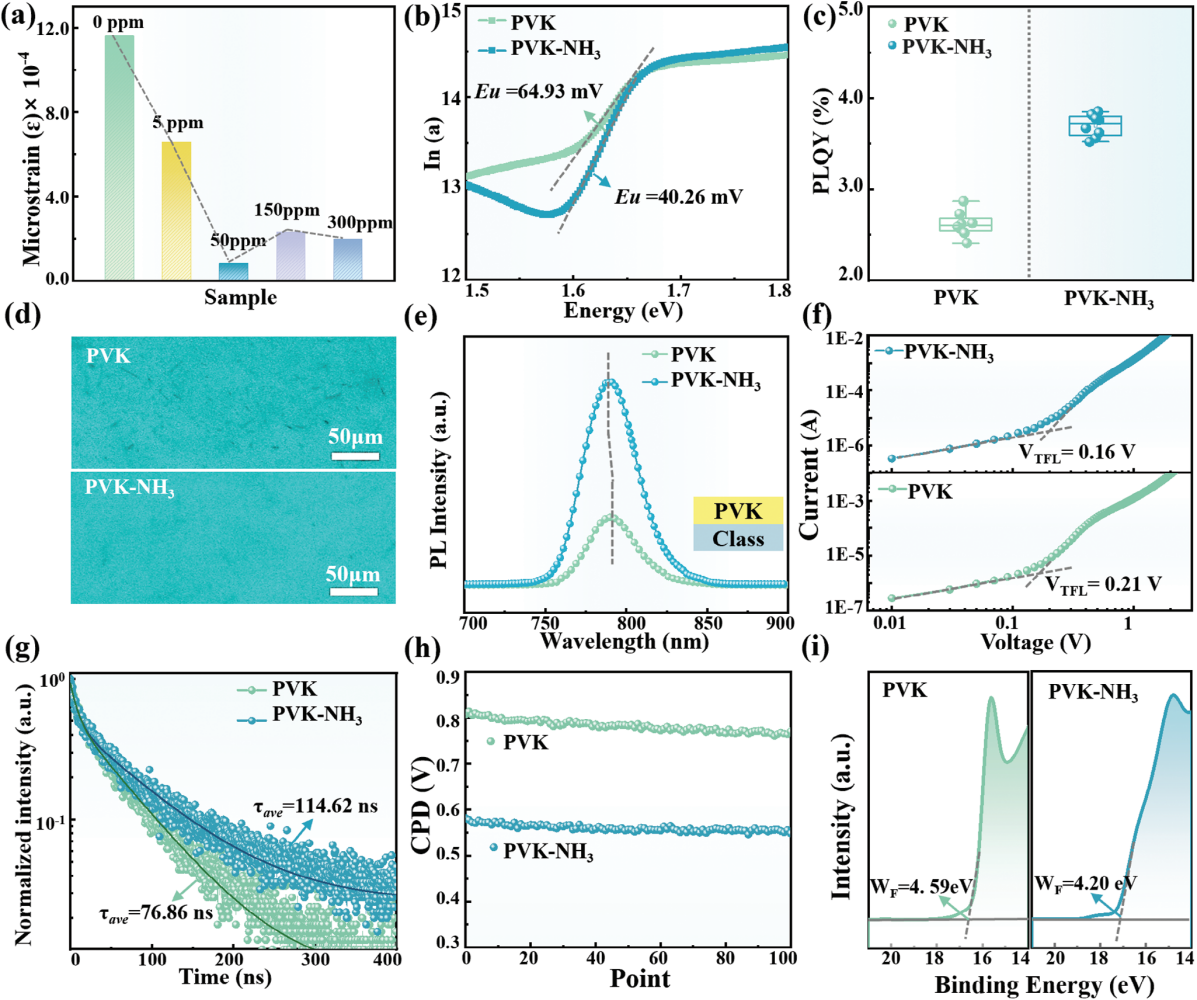
a) The statistics of ɛ values of PVK films treated with different concentrations of NH_3_ gas. b) Absorption coefficient derived from the UV–vis absorption spectra versus energy for PVK and PVK‐NH_3_ films. c) The values of PLQY of PVK and PVK‐NH_3_ films under photoexcitation of 520 nm. d) The CLSM images of PVK and PVK‐NH_3_ films. e) The PL spectra of PVK and PVK‐NH_3_ films on class substrates under surface irradiation. f) J‐V curves of SCLC test from electron‐only devices. g) TRPL spectra of PVK and PVK‐NH_3_ films deposited on the bare glass. h) The CPD curves of PVK and PVK‐NH_3_ films. i) The *W_F_* of PVK film before and after treatment with NH_3_ gas.

To further evaluate the impact of NH_3_ treatment, XRD patterns of PVK films exposed to varying NH_3_ concentrations, treated temperatures, and treated times were investigated (Figure S21, Supporting Information). When treated with different conditions, the characteristic peak of PbI_2_ in PVK exhibited the lowest intensity at a gas concentration of 50 ppm at 100 °C for 10 min, suggesting the most effective in NH_3_ passivating residual PbI_2_ under this condition. Meanwhile, compared to the pristine PVK, a slight increase in the intensity of the main diffraction peak of NH_3_ treated PVK was observed, indicating an improvement in the crystallinity of the PVK film (Figure S21a, Supporting Information), consistent with the SEM discussion results. Using XRD spectra of PVK‐NH_3_ films, the influence on the ɛ in PVK films treated with different NH_3_ concentrations was further examined. Based on the Lorentz function, Williamson‐Hall plots were achieved (Figure S22a−e, Supporting Information).^[^ ^39^ ^]^ The ɛ of PVK‐NH_3_ films could be derived from the corresponding slopes of the achieved linear function.^[^ ^39^ ^]^ The summarized ɛ values of PVK‐NH_3_ films are exhibited in **Figure** **4**a. As the NH_3_ concentration increases, the value of ɛ decreases first, reaching a minimum value of 0.82 × 10^−4^ at the optimal concentration of 50 ppm NH_3_. This demonstrates that appropriate NH_3_ gas treatment could effectively alleviate the residual ɛ in the PVK film, enhancing phase stability, and thus improving the quality of PVK crystals.^[^ ^40^, ^41^ ^]^

Then, the specific effect of NH_3_ gas treatment on the quality of PVK film was examined. Normally, the Urbach energy (*E_u_*) value, related to the loss in V_OC_, serves as a reliable indicator for evaluating the quality of PVK films. It can be extracted from the exponential decay of the absorption tail in the long wavelength edge region (1.58−1.61 eV) with characteristic energy.^[^ ^42^ ^]^ The reciprocal of the slope value of the curve depicted in Figure 4b represents the value of *E_u_*, which was identified as 40.26 mV for the PVK‐NH_3_ film and 64.93 mV for the PVK film. A reduced *E_u_* value suggests lower electronic barriers and suppression of intrinsic defects within the band tails,^[^ ^43^ ^]^ leading to films with superior crystallinity and reduced trap density. To assess the above effects, the photoluminescence quantum yield (PLQY) and confocal laser scanning microscope (CLSM) images of PVK and PVK‐NH_3_ films were compared (Figure 4c,d). A noticeable enhancement in the PLQY of PVK‐NH_3_ films could be observed when comparing the PLQY statistical values of films pre and post NH_3_ gas treatment. In line with the PLQY enhancement, the black regions in the CLSM images were minimized and brightness was enhanced, signifying a substantial reduction in luminescent defects, further corroborating the improved crystal quality of the film.

The effect of NH_3_ treatment on the carrier dynamics of PVK films was qualitatively analyzed through PL spectroscopy. Initially, the PL spectra of PVK films pre and post NH_3_ treatment as well as under different treat conditions were obtained (Figure 4e; Figure S23a–c, Supporting Information). In comparison to the pristine PVK thin film, the intensity of PL peak was markedly increased and the peak position was blue‐shifted from 790.2 to 789.9 eV. This provides further evidence for the passivation effect of NH_3_ on inherent defects on the PVK surface. The enhancement in peak intensity signified a reduction in defect states that hinder carrier transport at the interface, then effectively enhancing carrier extraction.^[^ ^44^ ^]^ In addition, the SCLC test with the electronic device configuration of ITO/ETLs/PVK/PCBM/Ag was utilized to quantitatively evaluate the N_t_ of PVK, as shown in Figure 4f. The N_t_ values, calculated based on the obtained V_TFL_ values of 0.21 and 0.16 eV, were determined to be 6.06 × 10^15^ and 4.62 × 10^15^ cm^−3^, respectively. The decrease in N_t_ value was attributed to the effective targeted passivation of uncoordinated Pb^2+^ and V_I_ defects on the surface of PVK‐NH_3_ film, thereby promoting carrier transport and extraction.

The TRPL spectroscopy was employed to further investigate the carrier transport in PVK after NH_3_ surface treatment. The specific fitting time constants were recorded in Table S5 (Supporting Information). The TRPL spectra of the PVK and PVK‐NH_3_ films exhibit an increased carrier transport lifetime from 76.86 to 114.62 ns with NH_3_ treatment (Figure 4g). This indicates more effective carrier transport at the interface. Moreover, NH_3_ gas treatment also lowered the CPD of the PVK film, symbolizing a decrease in *W_F_* (Figure 4h). Subsequently, the *W_F_* values of PVK and PVK‐NH_3_ films were determined to be 4.59 and 4.20 eV using UPS, respectively. This result further confirms that the NH_3_ treatment on the PVK surface effectively reduced the *W_F_* value, which is conducive to the reduction of the carrier transport barrier and further facilitates carrier transport (Figure 4i).

**Figure 5:**
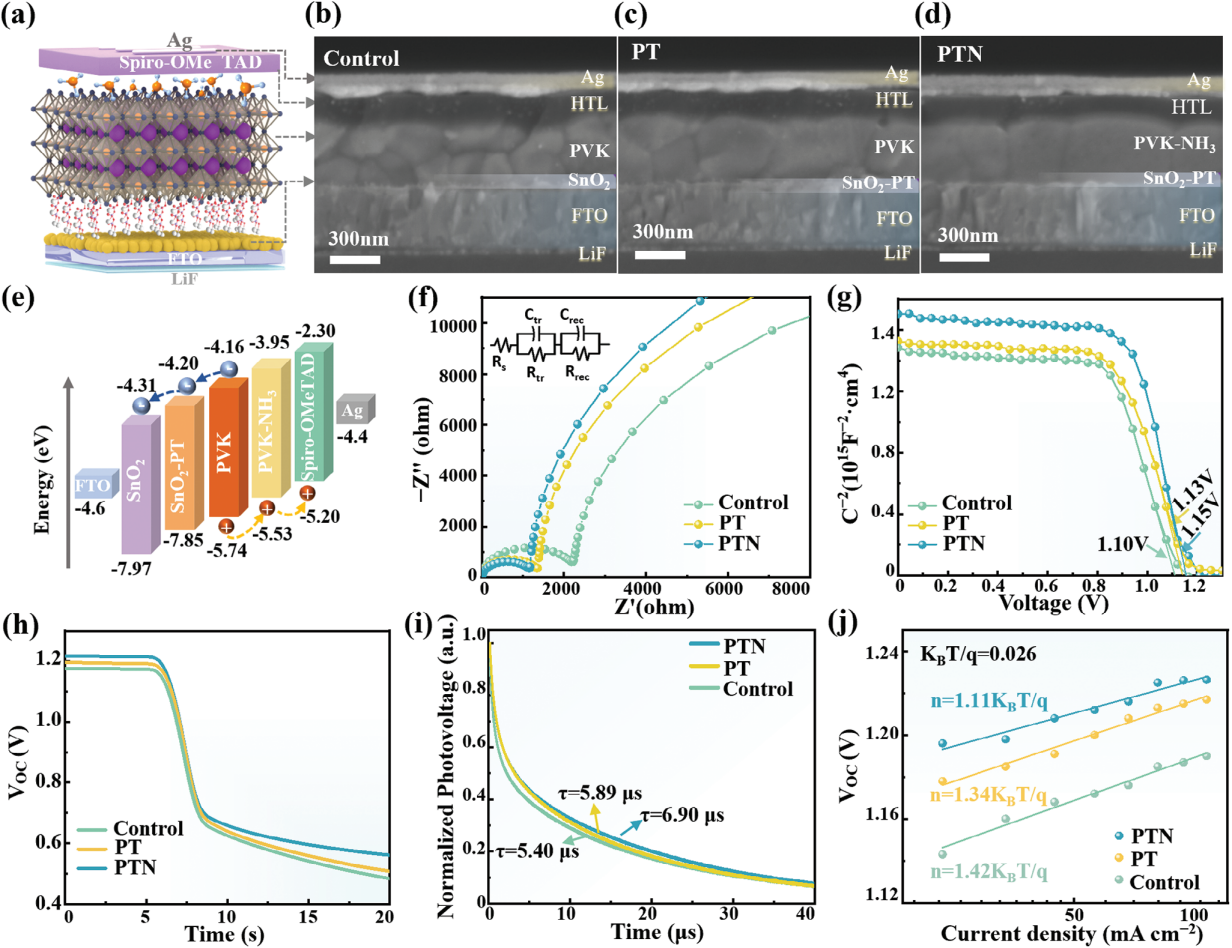
a) Device structure of PSCs with the passivation of inorganic PT and NH_3_ molecules. b−d) Cross‐section SEM images of Control, PT, and PTN. e) The energy level alignment of PSCs modified by inorganic PT molecules and NH_3_ gas. f) EIS plots of PSCs measured in the dark. g) Mott Schottky, h) OCVD curves, i) TPV curves, and j) V_OC_ dependence on light intensity measurements of different types of PSCs.

To investigate the efficacy of a gas‐assisted all‐inorganic dual‐interface passivation strategy, the XRD and UV absorption tests were first conducted with SnO_2_/PVK film as a control sample (Figures S24 and S25, Supporting Information). Following dual‐interface treatment, there is a decrease in the PbI_2_ diffraction peak intensity and an enhancement of absorption intensity in UV spectra, respectively. These results indicate that the as‐designed dual‐interface passivation strategy via inorganic PT and NH_3_ molecules further improved the quality of PVK films, which is beneficial for enhancing the J_SC_ of the following PSC device. Then, the PSCs with a basic device structure of LiF/FTO/SnO_2_/PVK/Spiro‐OMeTAD/Ag were prepared to further evaluate the impact of the dual inorganic small molecule passivation strategy. Here, for ease of description, the device optimized by inorganic PT molecules on the bottom interface was denoted as PT, while the device synergistically optimized by inorganic PT molecules and NH_3_ gas both on the dual interfaces was represented as PTN (**Figure** **5**a). Figure 5b−d shows the corresponding cross‐sectional scanning SEM images of the overall device. In comparison, the PTN device exhibited a gradual transition in grain distribution from a smaller layered arrangement to a single layer with larger grains permeating the entire PVK film. The test result indicates that the synergistic effect of PT and NH_3_ could effectively enhance the longitudinal crystallinity of grains.

Figure 5e shows the overall energy level arrangement of the devices. The E_g_ values of SnO_2_ and SnO_2_‐PT films were determined by UV–vis spectroscopy and Tauc plot method (Figure S26a,b, Supporting Information). The valence band maximum (VBM) values based on UPS testing of SnO_2_ and SnO_2_‐PT were determined to be −7.97 and −7.85 eV, from which the CBM values were calculated to be −4.32 and −4.19 eV, respectively (Figure S26c,d, Supporting Information). The CBM of SnO_2_‐PT is closer to that of PVK, lowering the electron transport barrier, which not only promotes electron carrier transport but also helps to prevent electron carrier buildup on the SnO_2_ surface. Similarly, the VBMs of PVK and PVK‐NH_3_ films were determined to be −5.57 and −5.53 eV, respectively (Figures S27 and S28, Supporting Information). The VBM of PVK‐NH_3_ is closer to the VBM of Spiro‐OMeTAD, which is more conducive to hole carrier transfer at the interface and effectively inhibits the reverse transfer of electrons, reducing the NRR at the upper interface. Therefore, based on the NH_3_‐assisted all‐inorganic dual‐interface passivation strategy, a favorable energy level alignment was achieved, directly improving the carrier transport loss.

The aforementioned good interfacial crystal quality and favorable energy level arrangement provide an important incentive to improve the device performance. Therefore, we further analyzed the device performance as follows. Figure 5f presents the electrochemical impedance spectroscopy (EIS) data, which reveals the carrier transfer and recombination at the interface of the overall PSCs. An equivalent circuit model, consisting of a resistor (R_s_), a transport resistance (R_ct_) in the high‐frequency region, and a series resistance (R_rec_) in the low‐frequency region, was employed to fit the data (inset of Figure 5e). It's apparent that the PTN device has smaller R_ct_ values and larger R_rec_ values compared to the control and PT devices. This suggests a higher transfer rate and a lower recombination rate of charge carriers in PTN devices, which is advantageous for the FF of PSCs.^[^ ^45^ ^]^ A Mott Schottky curve was also measured, as illustrated in Figure 5g. The built‐in potential (V_bi_) of PTN devices has risen from a controlled 1.10 V to an optimized 1.15 V, providing a stronger driving force for the separation of photo‐generated charge carriers and electron‐hole pairs, thereby enhancing the V_OC_ in PSCs.^[^ ^46^ ^]^ Open circuit photovoltage decay (OCVD) measurement was further utilized to reveal the internal carrier transport and recombination states of PSCs (Figure 5h). Owing to carrier transport equilibrium, the V_OC_ decay rate over time is typically influenced by carrier transport equilibrium‐related space charge complexation.^[^ ^47^ ^]^ Thanks to the good interface quality in PTN devices, the PTN device with a V_OC_ of 1.229 V exhibits a slower photovoltage decay time compared to the other two devices.

Additionally, transient photovoltage (TPV) measurements were conducted to detect the recombination lifetime of carriers. As displayed in Figure 5i, the significantly extended photovoltage decay lifetime further demonstrates the suppressed trap‐assisted recombination due to the inorganic molecules passivation, especially in the PTN device.^[^ ^48^ ^]^ Figure 5j shows the dependence curve of V_OC_ on the light intensity of various PSCs. The ideal factor (n) is one of the most powerful evidence to describe carrier recombination in PSCs,^[^ ^49^ ^]^ which can be derived from the slope of the linear fitting in Figure 5i. The n values from both of the PT and PTN devices are lower than that of the control one. As expected, the lowest n value is from the PTN device (1.11 K_B_T/q), significantly lower than the 1.42 K_B_T/q of the control one. This strongly attests to the effectiveness of the dual‐interface synergistic passivation strategy in significantly suppressing defect‐assisted carrier recombination. The reduction in the carrier recombination process was further confirmed through dark current measurements (Figure S29, Supporting Information). Compared to the control device, the PT and PTN devices exhibited a decrease in reverse saturation current and conduction voltage. This indicates that more photocurrent directly passes through the cell without too many shunt paths,^[^ ^50^ ^]^ resulting in a reduction in leakage current. This conclusion aligns with the aforementioned analysis.

**Figure 6:**
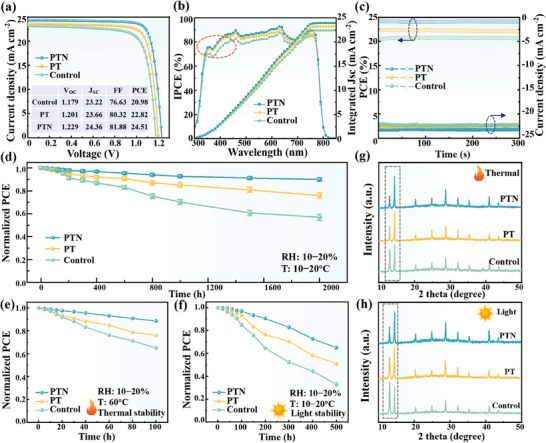
a) J‐V curves of the corresponding PSCs and their main parameters (inset table). b) IPCE and integrated J_SC_ of the corresponding PSCs. c) The steady current density of the corresponding PSCs. d) Normalized PCE of the PSCs stored in an environment with 10−20% RH for 2000 h at 10−20 °C. e) Normalized PCE of the PSCs stored in an environment with 10−20% RH for 100 h at 60 °C. f) Normalized PCE of the PSCs under continuous light irradiation at AM1.5 G, 100 mW**·**cm^−2^ for 500 h. g−h) XRD patterns of aged Control, PT, and PTN films under thermal and light conditions.

The detailed photovoltaic parameters obtained from the current density voltage (J‐V) curves of the corresponding devices are summarized in the inserted table in **Figure** **6**a. As compared with the control device, the synergistic effect of dual‐interface passivation strategy via inorganic PT and NH_3_ molecules resulted in significantly increased V_OC_ (from 1.179 to 1.229 V) and FF (from 76.63% to 81.88%), finally leading to an increase in PCE from 20.98% to 24.51%. This is the highest PCEs based on MA‐contained organic‐inorganic hybrid PSCs, as shown in Table S6 (Supporting Information). Additionally, the statistical distribution of the photovoltaic parameters of devices prepared under different conditions was depicted in Figures S30 and S31 and Table S7 (Supporting Information), aligning with the aforementioned conclusion of gas concentration optimization. Figure 6b presents the incident photon‐to‐electron conversion efficiency (IPCE) of the three PSCs. The enhanced photoresponse of the PTN device in the range of 350−450 nm indicates fewer disordered interfacial states between PVK‐NH_3_ and SnO_2_‐PT, enabling efficient photoelectron collection by the electrodes.^[^ ^51^ ^]^ The integrated J_SC_ of the corresponding devices were determined to be 22.41, 23.23, and 23.90 mA·cm^−2^, respectively, consistent with the values obtained from the current‐voltage (J‐V) curve. Moreover, the J_SC_ value was further corroborated by the current‐time (I‐T) curve over 600 s under continuous light radiation at the maximum power point, as illustrated in Figure 6c. A PCE of 24.19% was achieved for the PTN device biased at 1.04 V, whereas the control and PT devices yield 20.51% and 22.39% respectively, and these values are close to those obtained from the J‐V curves. Moreover, the forward and reverse J‐V scans were depicted in Figures S32a–c (Supporting Information). The hysteresis index decreased from 6.62% of the control device to 2.89% of the PTN device (Table S8, Supporting Information). This can be attributed to the effective passivation and ɛ relief from the synergistic effect of the dual‐interface passivation strategy via inorganic PT and NH_3_ molecules.

The stability of PSCs remains the most challenging issue limiting their commercialization.^[^ ^52^ ^]^ The analysis of the above test results shows that by applying the dual‐interface passivation strategy via inorganic PT and NH_3_ molecules, the PTN device not only achieved high‐quality upper and bottom interfaces but also effectively relieved film ɛ and enhanced the crystal quality of the PVK film. All these properties are very facilitative to achieve good stability. Therefore, the long‐term stability, thermal stability, and light stability of unpackaged devices are further depicted in Figure 6d−f. As shown in Figure 6d, the PTN device could maintain 90% of its initial efficiency after 2000 h of long‐term aging in air, significantly outperforming the PT (76%) and control devices (57%), since the hydrophobicity of PVK thin films has been enhanced through dual‐interface optimization (Figure S33, Supporting Information). To better assess the impact of the dual‐interface passivation strategy on device thermal stability, PTAA was selected as the hole transport layer (HTL) for this test. The device parameters of various devices prepared using PTAA as the HTL were summarized in Table S9 (Supporting Information). Under conditions of relative humidity (RH) of 10−20% and temperature of 60 °C (Figure 6e), the PTN device retained 88% of its initial PCE value after aging for 100 h, whereas the control device could only maintain 64%. Subsequently, the devices were exposed to sunlight (AM 1.5G, 100 mW·cm^−2^) under a nitrogen atmosphere for 500 h. The photostability of the PTN device preserved 65% of the initial performance, also much higher than those of the control and PT devices (Figure 6f).

Furthermore, to better evaluate the aging rate of the devices, XRD patterns of various types of PVK films under different conditions were measured (Figure S34, Supporting Information). The enhanced stability of the devices was mainly validated by monitoring the change in the peak intensity ratio of PbI_2_ to the main diffraction peak at the (100) crystal plane of PVK. As summarized in Table S10 (Supporting Information), the ratio of PTN films is always significantly lower than that of control and PT films regardless of the aging conditions. The above analysis suggests that the PTN device demonstrated superior environmental, thermal, and light stability compared to the control or PT devices. The improved device stability was primarily attributed to the effective passivation of inorganic PT molecules and NH_3_ gas in an all‐around way. Through the proposed dual‐interface passivation strategy which could effectively avoid solid‐liquid contact, significantly minimize defect sites at the interface of the device, thereby reducing the water‐oxygen intrusion channels.

## 3 Conclusion

In summary, we adopted an NH_3_ gas‐assisted all‐inorganic dual‐interface passivation strategy to achieve stable and non‐destructive defect passivation at the entire interfaces of the PSCs. On the one hand, the introduction of PT at the bottom interface filled the O_V_ and tuned the *W_F_* of SnO_2_, passivated the uncoordinated Pb^2+^ at the bottom of PVK, and promoted the longitudinal growth of PVK crystals. On the other hand, the NH_3_ gas treatment passivated the uncoordinated Pb^2+^ on the PVK surface, increased the formation energy of V_I_ from 1.17 to 1.54 V, and alleviated the ɛ of PVK film. The reduction of the above dual‐interface defect states effectively promoted carrier transport at the interfaces. More excitingly, the PVK surface passivation with NH_3_ overcame the drawbacks of conventional surface treatments using solvents, such as the reconstruction of PVK surface composition and the introduction of uncertain defect types and quantities. Ultimately, based on the dual‐interface synergistic effect, the PCE of PSCs contained MA reached 24.51% (0.1 cm^2^), while the V_OC_ and FF were as high as 1.229 eV and 81.88%, respectively. The long‐term stability of unpackaged optimized devices remained at 90% of the initial PCE after 2000 h of aging, while also exhibiting good thermal and photo stability. This study provides a simple and efficient pure gas‐assisted inorganic molecular dual‐interface passivation strategy to promote the commercial development of PSCs.

## Conflict of Interest

The authors declare no conflict of interest.

## Supporting information

Supporting Information

## Data Availability

The data that support the findings of this study are available from the corresponding author upon reasonable request.
