# Supplementary material for: Gas Molecule Assisted All‐Inorganic Dual‐Interface Passivation Strategy for High‐Performance Perovskite Solar Cells
Source: Adv Sci (Weinh). 2024 Jul 4;11(34):2404444. doi: 10.1002/advs.202404444 (PMC11425670; doi:10.1002/advs.202404444)
Supplement: Supplementary file 1 — Supporting Information [file ADVS-11-2404444-s001.docx]

**Gas Molecule Assisted** **All-Inorganic Dual-Interface Passivation Strategy for High-Performance Perovskite Solar Cells**

Fancong Zeng^a^, Lin Xu*^a,c^, Jiahe Xing^a^, Yanjie Wu^a^, Yuhong Zhang^a^, Huan Zhang^a^, Chencheng Hu^a^, Biao Dong^a^, Xue Bai^a^, Hongwei Song*^a,b^

^a^State Key Laboratory on Integrated Optoelectronics, College of Electronic Science and Engineering, Jilin University, 2699 Qianjin Street, Changchun, 130012, PR China

^b^School of Physics and Electronics, Henan University, Kaifeng, 475001, PR China

^c^Key Laboratory of Bionic Engineering, Ministry of Education, College of Biological and Agricultural Engineering, Jilin University, Changchun 130022, PR China

*Corresponding author

E-mail addresses: [linxu@jlu.edu.cn](mailto:linxu@jlu.edu.cn) (L. Xu), [songhw@jlu.edu.cn](mailto:songhw@jlu.edu.cn) (H. Song)

**Experimental Section**

**Materials**

Potassium tripolyphosphate (PT) (≥95%) was purchased from Shanghai McLean Reagent Co., Ltd. Ammonia (NH_3_) was purchased from Juyang Co., Ltd. Tin oxide (IV) (SnO_2_) (15% H_2_O colloidal dispersion) was purchased from Alfa Aesar. Ultra-pure water, acetonitrile, N, N-dimethylformamide (DMF) (>99.0%), dimethyl sulfoxide (DMSO) (>99.5%), and chlorobenzene (CB) (99.9%) were all purchased from Sigma Aldrich. Cesium iodide (CsI) (≥99.99%), formamidine iodide (FAI) (>99.5%), methyl ammonium bromide (MABr) (>99.5%), Methylamine chloride (MACI) (>99.5%), lead bromide (PbBr_2_) (>99.99%), lead iodide (PbI_2_) (>99.99%) and Spiro-OMeTAD were all purchased from Xi'an Yuri Solar Co, Ltd. (China). FTO was purchased from YouXuan Trade Co., Ltd. (China).

**Preparation of precursor solution**

The precursor solution of tin dioxide was obtained by mixing SnO_2_ colloid with ultrapure water in a volume ratio of 1:3. The different quality PT powders were dissolved in 1mL of ultrapure water to obtain PT solutions of different concentrations. The precursor solution of Cs_0.05_(FA_0.90_MA_0.10_)_0.95_Pb(I_0.90_Br_0.1_)_3_ (CsFAMA) PVK was obtained by mixing FAI (205.6 mg), MABr (14.8 mg), CsI (18.2 mg), MACI (16 mg), PbBr_2_ (26.5 mg), and PbI_2_ (642.9 mg) in a mixed solvent of DMF and DMSO (volume ratio=4:1). Spiro-OMeTAD precursor solution for spin-coating hole transport layers were obtained by adding 72.3 mg of Spiro-OMeTAD, 28.8 μL of 4-tert-butylpyridine (tBP), and 17.5 μL of Li-bis-(trifluoromethanesulfonyl) imide solution (520 mg Li-TFSI in 1 mL of acetonitrile) to a 1 mL CB.

**Device fabrication**

The FTO substrates were cleaned sequentially in detergent, deionized water, acetone, and ethanol in ultrasonic bath for 15 min, respectively. The cleaned FTO substrates were treated with ozone for 30 min before depositing SnO_2_ film. The precursor solution of SnO_2_ or SnO_2_-PT ETL was deposited on the FTO substrate with spin coating at 5000 rpm for 30 s, and then annealed in air at 150°C for 30 min. The CsFAMA thin film was coated on ETL through two consecutive spin coating programs at 1000 rpm for 10 s and 5000 rpm for 30 s, respectively. At the beginning of the second step, 130 μL CB was quickly added in the matrix, and then annealed at 100°C for 1 h to obtain the CsFAMA thin film. For NH_3_ treatment, the obtained CsFAMA films were placed in different concentrations of NH_3_ gas in a sealed glass bottle. The NH_3_ used comes from an ordered 10,000 PPM NH_3_ cylinder. The volume of the sealed container used is 500 mL. The different concentrations of NH_3_ could be obtained by adjusting the volume of NH_3_ injected into the sealed container. It is important to note that the entire process of injecting NH_3_ takes place in a glove box. Then, the Spiro-OMeTAD was deposited onto the prepared PVK film at 3000 rpm for 30 s as the hole transport layer. Finally, a 100 nm of Ag electrode was thermally deposited under vacuum conditions. On the back side of FTO, a 3 nm LiF was thermally deposited as the anti-reflection layer. The effective area of the device is 0.1 cm^2^.

**Characterization**

The top and cross-section SEM measurements were performed by a SIRION field-emission SEM with an EDS system (Nova Nano SEM 430). The roughness and the AFM of the film were characterized by atomic force microscopy (AFM, Dimension Fastscan Bio, CA). The XPS data were measured with an Al Kα monochromatized source (ESCALAB MARK II, VG Inc.). The Raman spectroscopy was performed with Lab RAM HR Evolution (Jobin-yvon, Horiba, France) excited by a He-Ne laser with a wavelength of 532 nm. The XRD patterns were conducted via a Rigaku TTR III X-ray diffractometer (Tokyo, Japan) using a monochromatized Cu target as radiation source in the 2θ range of 10–80°. Time of flight secondary ion mass spectrometry (ToF-SIMS) for detecting ion distribution. The UPS was measured under UHV with a helium discharge lamp (hν=21.22 eV). The PL spectra were performed by the Omni-λ300 Monochromator/Spectrometer of Zolix. To detect the TRPL trace, the excitation source is a Nano-LED working with a wavelength of 482 nm and a pulse duration <200 ps, and the signal is recorded at 767 nm by the time-correlated single-photon counting detection technique. The optical absorption spectra were carried out by using a UV-1800 spectrometer. Fourier transform infrared spectroscopy (FTIR) data obtained through FTIR (Nicolet iS10). The absolute PLQYs of the perovskite films were measured on a fluorescence spectrometer (FLS920P, Edinburgh Instruments) equipped with an integrating sphere. Laser scanning confocal microscopy (LSCM) was performed with 405 nm excited (OLYMPUS, FV1000). Transient photovoltage (TPV) decay curves were obtained using the home-made CEL-TPV1000 device produced by Beijing Zhongjiao Jinyuan Technology Co. Ltd. The OCVD was measured with Princeton Applied Research. The EIS was obtained with an impedance analyzer (Solartron CHI660C). The Nyquist plots and EIS parameters were fitted using the software Z-View. The dark current, light intensity voltage curve and current steady state of perovskite solar cells (PSCs) were collected on the Keithley 2400 source table, respectively. J-V characteristics of the PSCs were measured using a solar simulator (AM 1.5G, Zolix SS150 Solar Simulator) calibrated using a standard silicon solar cell. Keithley 2400 was used for the J-V scan by applying an external voltage bias and all PSCs were tested in the forward and reverse scan directions following standard procedures at a rate of 10 mV·s^−1^. The cells were treated with a black metal mask with an area of 0.1 cm^2^. Using an IPCE Measurement System (Zolix Solar Cell Scan 100) under DC mode, where the monochromatic beam was supplied by a Zolix LSP-T150 W Tungsten-Halogen Light Source. The monochromatic light intensity was adjusted using a reference silicon sample.

**Statistical Analysis**

The grain size distribution of thin film samples was measured using the Nano Measurer program to obtain a certain amount of grain size (50-200 grains) and a size summary report. The normal distribution of the summarized data was then plotted using the Origin program for statistical analysis. For device performance parameter statistics, firstly, the specific parameter values of 20 devices of each type were recorded, and then use the Origin program to draw a box statistical chart of the recorded data; For device stability statistics, different devices were tested 20 times under different conditions and the corresponding errors were calculated. Finally, the Origin program was used to draw a variation curve with error bars to analyze the stability of the devices under corresponding conditions.

**DFT calculation details**

The simulation analysis calculation was conducted within the density functional theory (DFT) framework of Quantum Espresso software packages (QE).^[1,2]^ The exchange-correlation energies were described using the generalized gradient approximation (GGA) with the Perdew-Burke-Ernzerhof (PBE) functional.^[3,4]^ The Projected augmented wave (PAW) method^[5]^ was employed for the pseudo-potentials of the H, C, N, Br, I, Cs and Pb-atoms. respectively, and the convergence criteria for the maximum force and energy oneach atom during structure relaxation were set to 0.02 eV·Å^−1^ and 10^−5^ eV.The Brillouin-zone sampling were conducted using Monkhorst-Pack (MP) grids of special points with the separation of 0.04 Å^−1^. For the structural optimization and the electronic structure calculations, respectively. The lattice parameters and atomic positions were relaxed with a convergence criterion for the total energy and the ionic forces set to 10^−5^ eV and 0.02 eV·Å^−1^, respectively. A semiempirical DFT-D3 force-field approach was used to include the physical Van Der Waals (VDW) interaction in our calculations.^[6,7]^ respectively, cut the surface and a vacuum space of 15 Å was adopted to avoid the interaction between the bounding layers.

**1. Binding energy**

Calculate the binding energy between the iodine vacancy (V_I_) and the iodide ions I^−^ and NH_3_ at the lattice, where the binding energy was calculated as follows:

E_b_=E_t1_−E_s1_−E_s2_

where E_t1_ is the total energy of the binding system, E_s1_ is the energy of the optimized clean surface slabs (with V_I_), and E_s2_ is the energy of the adsorbates (I^−^ and NH_3_) in vacuum.

Calculations show that the binding energy of NH_3_ with V_I_ (−1.09 eV) is greater than that of with I^−^ (−0.57 eV) to show that NH_3_ on the chalcopyrite surface interacts more favorably with V_I_ than with I^−^, resulting in stronger bonding with the uncoordinated Pb^2+^ sites in the Pb−I framework.

**2. Formation energy**

We calculated the formation energy of V_I_ on the surface of chalcogenide films before treatment with ammonia(1.17 eV); then we calculated the formation energy of V_I_ on the surface of chalcogenide films after treatment with ammonia(1.54 eV). It was found that V_I_ formation can be increased after NH_3_ treatment, where the vacancy formation energy was calculated as follows:

E_f_=E_v_−E_pref_+E_I_

where E_v_ denotes the total energy of the system containing one I vacancy, E_pref_ denotes the total energy of the system without defects, and E_I_ denotes the energy of one I atom.

DFT calculations were performed by Vienna Ab initio Simulation Package (VASP).^[1,2]^ PAW-PBE functionals were used to describe exchange-correlation effects between electrons.^[1,2]^ All structures were totally relaxed under energy convergence tolerance of $\text{1×}\text{10}^{-\text{5}}$ eV and stress lower than $\text{5×}\text{10}^{-\text{2}}$ eV·Å^−1^ with Monk horst Brillouin sampling mesh of$\text{4×4×2}$.^[3]^ The plane wave energy cutoff was set to be 600 eV for all calculations. In order to model surface of PVK, $\text{3×3}\text{ }$supercell was built and (110) plane was selected to be the plane of interest. Vacuum zone along z-direction was set to be 15 Å to avoid possible interaction between supercells.

**Figure captions**

**Figure S1**. (a) The chemical structure and (b) electrostatic surface potential (ESP) map of PT.

**Figure S2**. SEM images of pristine SnO_2_ (SnO_2_ 1-4) and SnO_2_-PT films (SnO_2_-PT 1-4).

**Figure S3**. Statistical distribution of grain size of pristine SnO_2_ (SnO_2_ 1-4) and SnO_2_-PT films (SnO_2_-PT 1-4).

**Figure S4**. The stylus profiler tests of pristine SnO_2_ films (9 samples).

**Figure S5**. The thickness variation curve of pristine SnO_2_ films (9 samples).

**Figure S6**. The stylus profiler tests of SnO_2_-PT films (9 samples).

**Figure S7**. The thickness variation curve of SnO_2_-PT films (9 samples).

**Figure S8**. EDS mapping of the Sn, O, P and K elements on the surface of the SnO_2_-PT film.

**Figure S9**. Sn 3d XPS spectra of SnO_2_ and SnO_2_-PT films.

**Figure S10**. (a) The transmittance and (b) I-V curves of SnO_2_-PT films modified with different concentrations of PT.

**Figure S11**. The SCLC of SnO_2_ and SnO_2_-PT films.

**Figure S12**. The *E_f_* of SnO_2_ and SnO_2_-PT films obtained by secondary-electron cut-off region of UPS spectra.

**Figure S13**. Hydrophobic angle of SnO_2_ and SnO_2_-PT films.

**Figure S14**. PT solubility in different solvents (The solution after PT dissolution is clear).

**Figure S15**. The electrostatic surface potential (ESP) map of NH_3_.

**Figure S16**. EDS mapping images and component distribution spectrogram of PVK and PVK-NH_3_ thin films.

**Figure S17**. AFM images of PVK-NH_3_ thin films.

**Figure S18**. The size distribution of PVK, PT-PVK, and PVK-NH_3_ thin films.

**Figure S19**. I 3d XPS spectra for the PVK and PVK-NH_3_.

**Figure S20**. (a, b) The adsorption states of NH_3_ on the (001) surface of PVK with V_I_ and I^−^ under DFT simulation. (c)The adsorption energy between NH_3_ and V_I_, I^−^ sites.

**Figure S21**. (a) XRD patterns of PVK films under different NH_3_ concentrations. (b, c) XRD patterns of PVK films treated at different temperatures and times based on the optimal NH_3_ gas concentration (50 ppm).

**Figure S22**. (a−e) The Williamson-Hall plots of PVK films with different NH_3_ treatment concentrations.

**Figure S23**. (a) PL spectra of PVK films treated with different concentrations of NH_3_. (b, c) Exploring the PL spectra of PVK films treated at different temperatures and times based on the optimal concentration of NH_3_ (50 ppm).

**Figure S24**. UV absorption spectra of Control, PT modification, and dual modification of PT and NH_3_ membranes films.

**Figure S25**. XRD patterns of Control, PT modification, and dual modification with PT and NH_3_ films.

**Figure S26**. (a, b) The (*ahv*)^2^ versus *hv* curves and (c, d) VBM of UPS spectra for SnO_2_ and SnO_2_-PT film.

**Figure S27**. (a, b) The (*ahv*)^2^ versus *hv* curve of PVK and PVK-NH_3_ films.

**Figure S28.** (a−d) The VBM and full spectrums of UPS spectra for PVK and PVK-NH_3_ films.

**Figure S29.** Dark J-V curves of the corresponding PSCs.

**Figure S30**. Statistics of PCE for the different concentrations of PT surface-treated SnO_2_ of 20 devices, separately.

**Figure S31**. Statistics of V_OC_, J_SC_, FF, and PCE for Control, PT, and different gas concentrations of 20 devices.

**Figure S32**. The forward and reverse scan J-V curves of Control, PT, and PTN PSCs.

**Figure S33**. (a−c) Hydrophobic angle images of Control, PT modification, and dual modification of PT and NH_3_ membranes.

**Figure S34**. Aging (80 days) XRD patterns of Control, PT, and PTN films without encapsulation under air conditions.

**
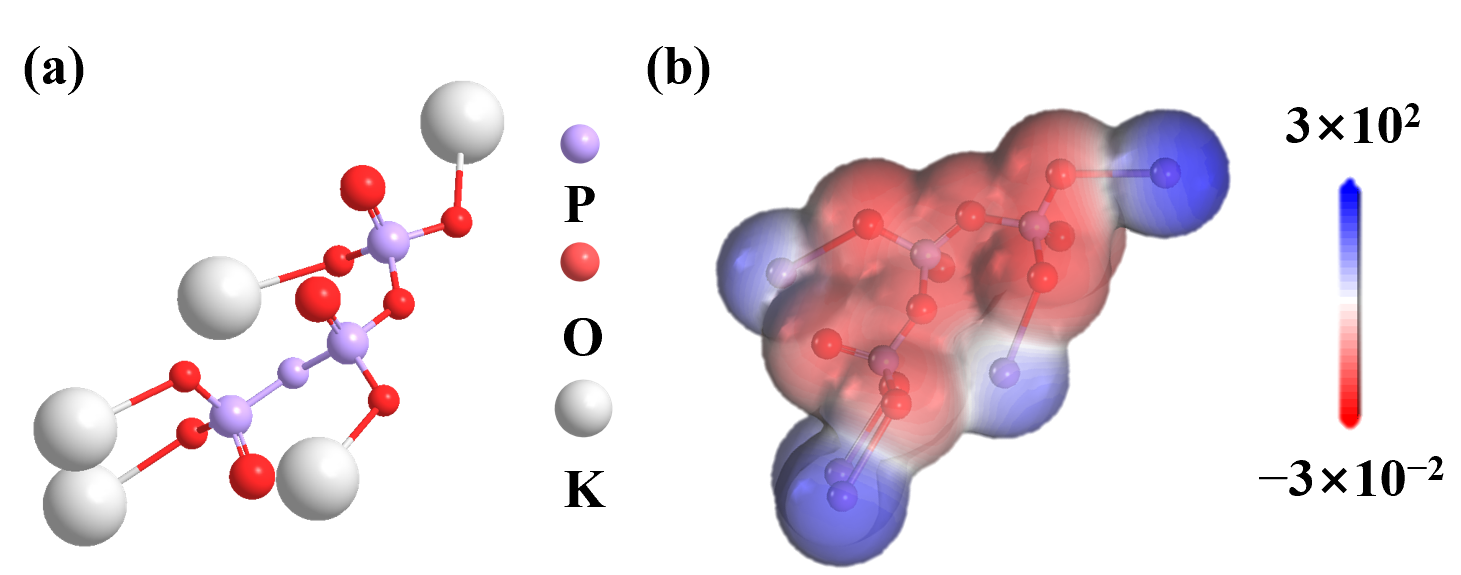
**

**Figure S1**. (a) The chemical structure and (b) electrostatic surface potential (ESP) map of PT.


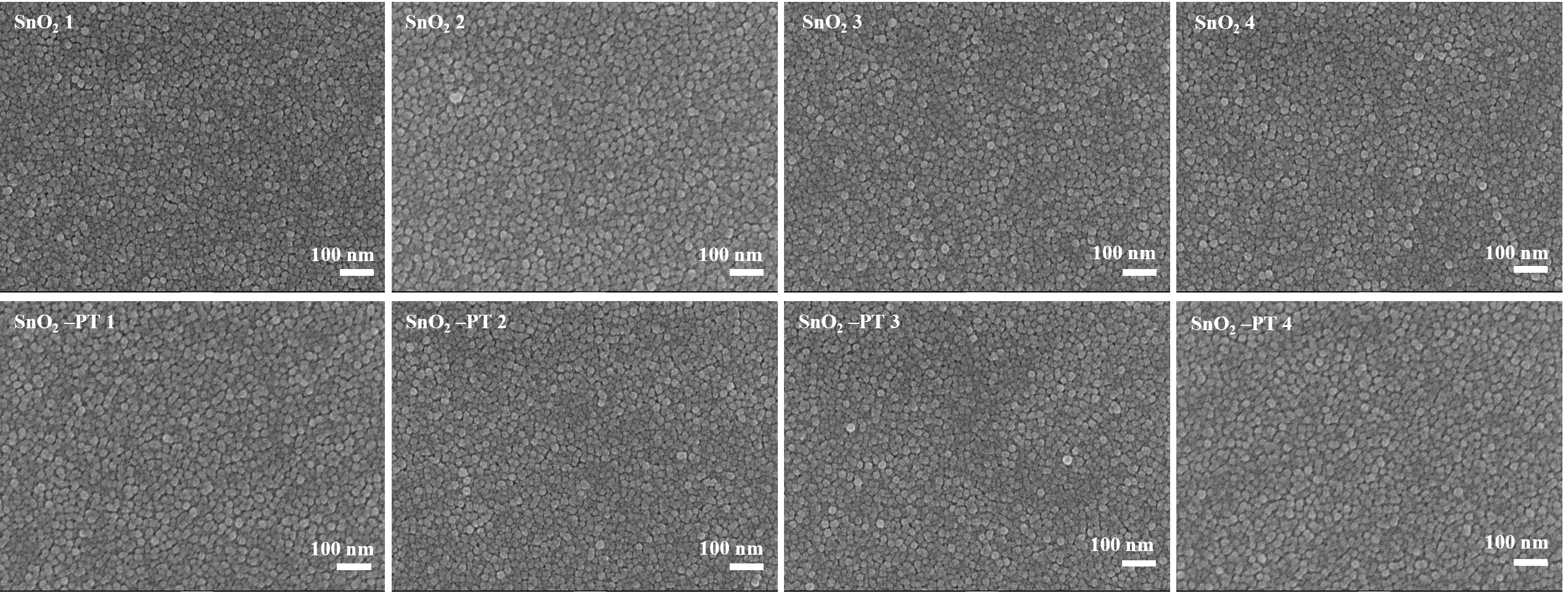


**Figure S2**. SEM images of pristine SnO_2_ (SnO_2_ 1-4) and SnO_2_-PT films (SnO_2_-PT 1-4).


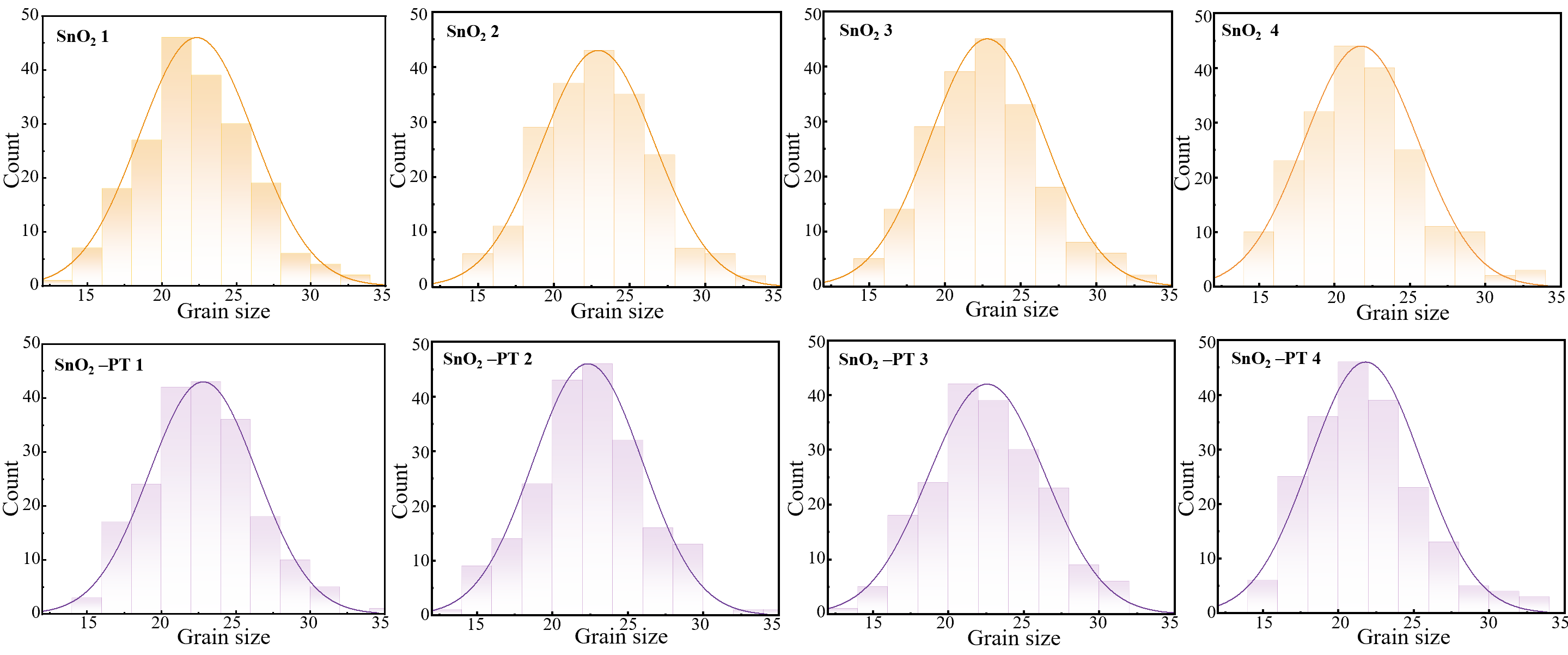


**Figure S3**. Statistical distribution of grain size of pristine SnO_2_ (SnO_2_ 1-4) and SnO_2_-PT films (SnO_2_-PT 1-4).


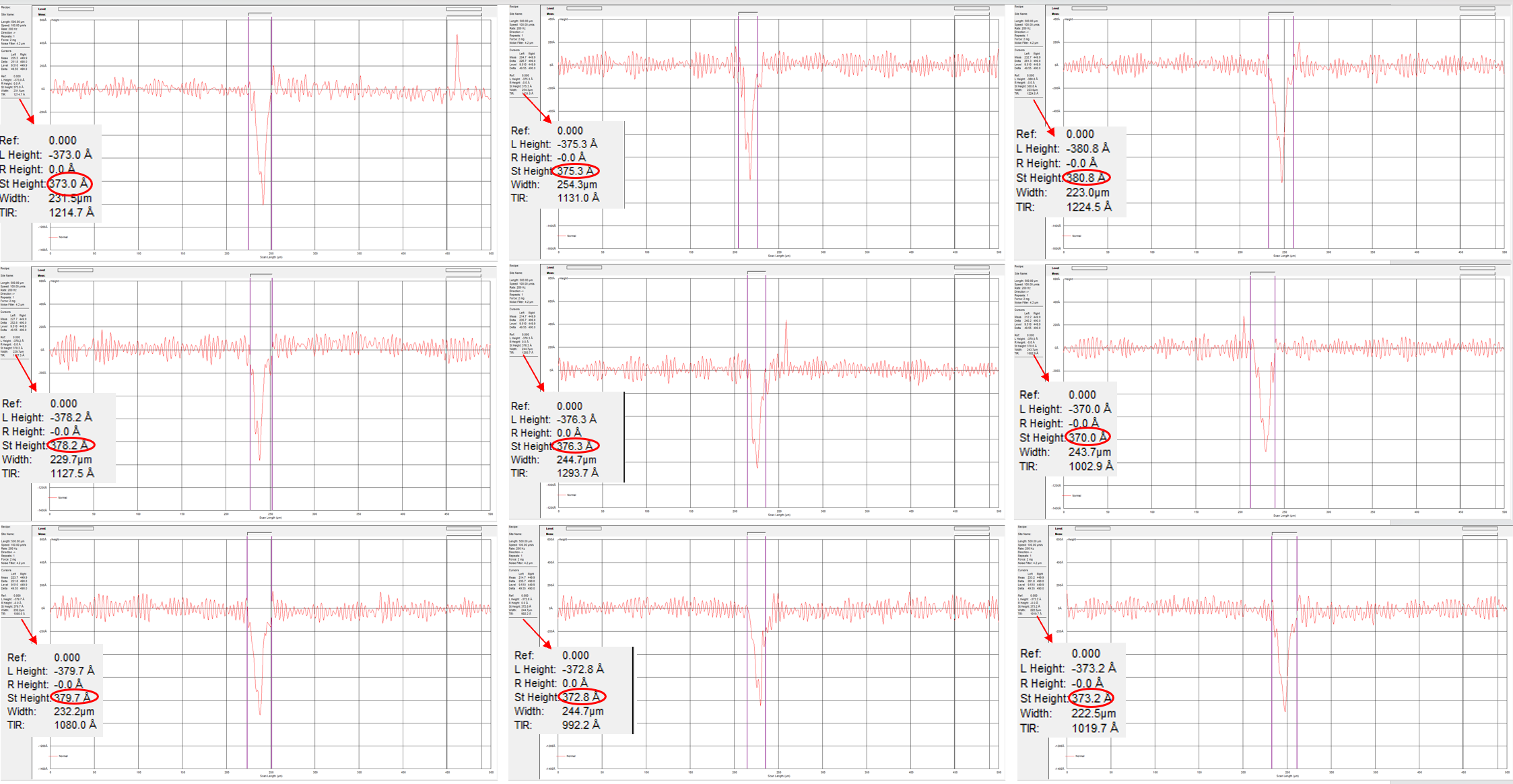


**Figure S4**. The stylus profiler tests of pristine SnO_2_ films (9 samples).


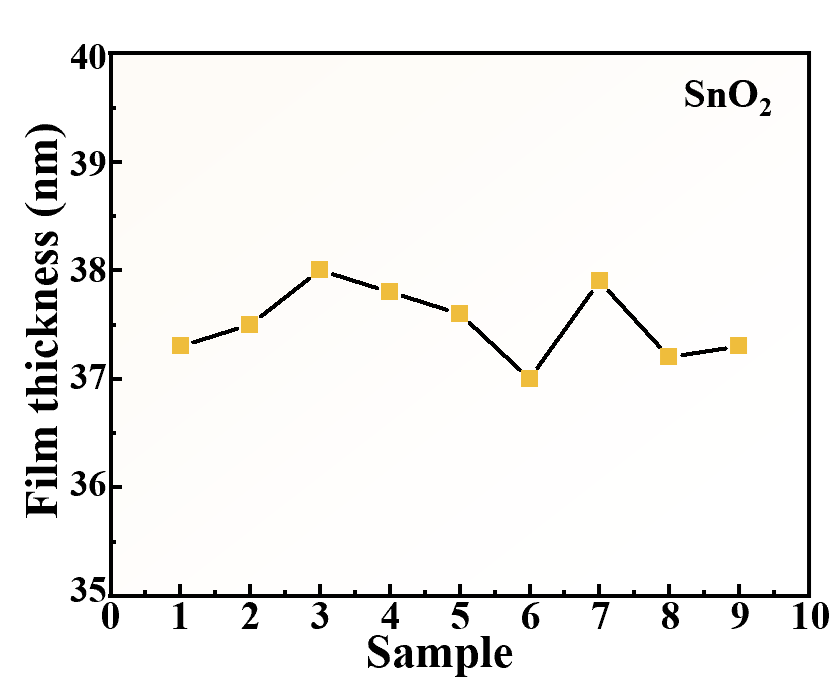


**Figure S5**. The thickness variation curve of pristine SnO_2_ films (9 samples).


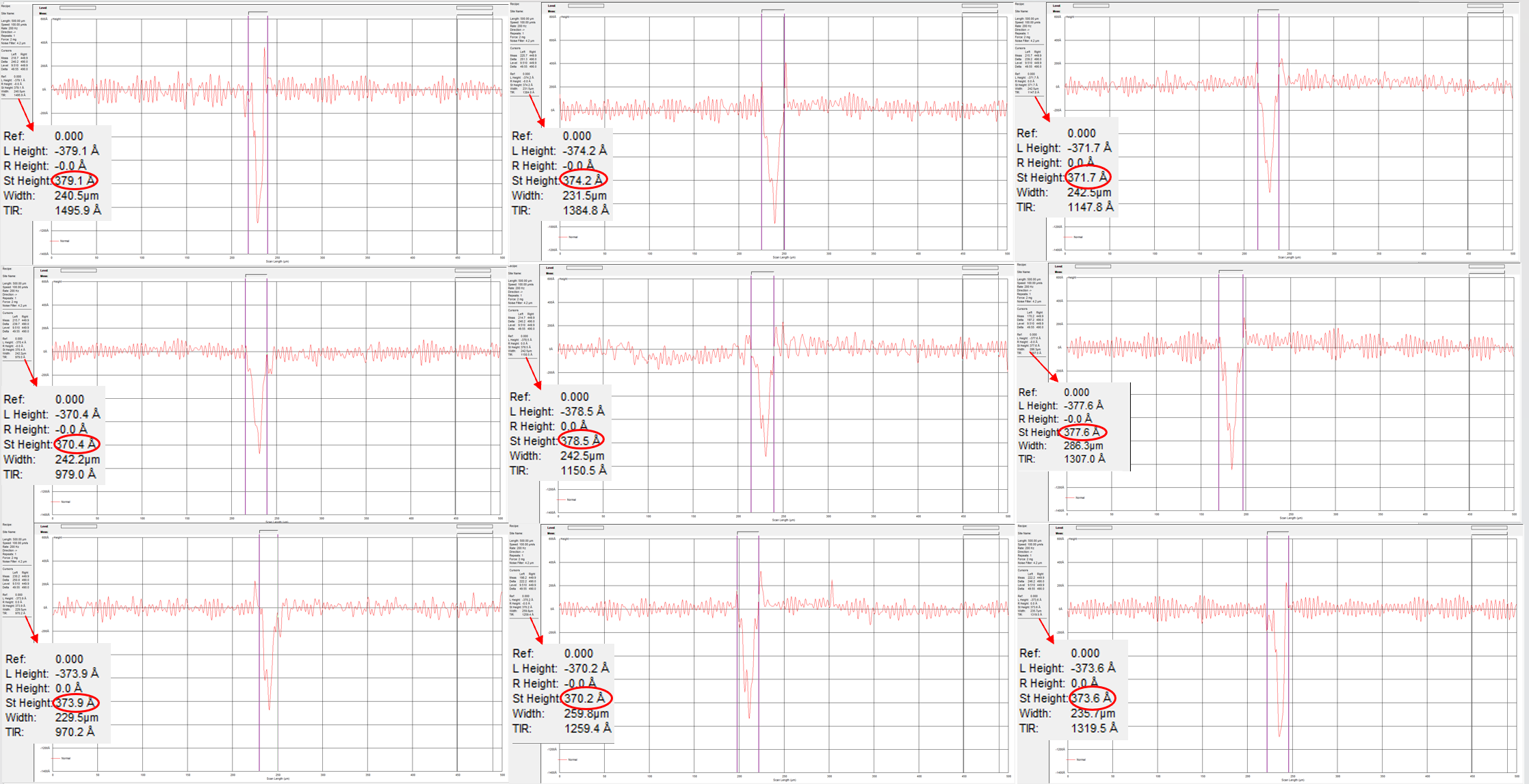
**Figure S6**. The stylus profiler tests of SnO_2_-PT films (9 samples).


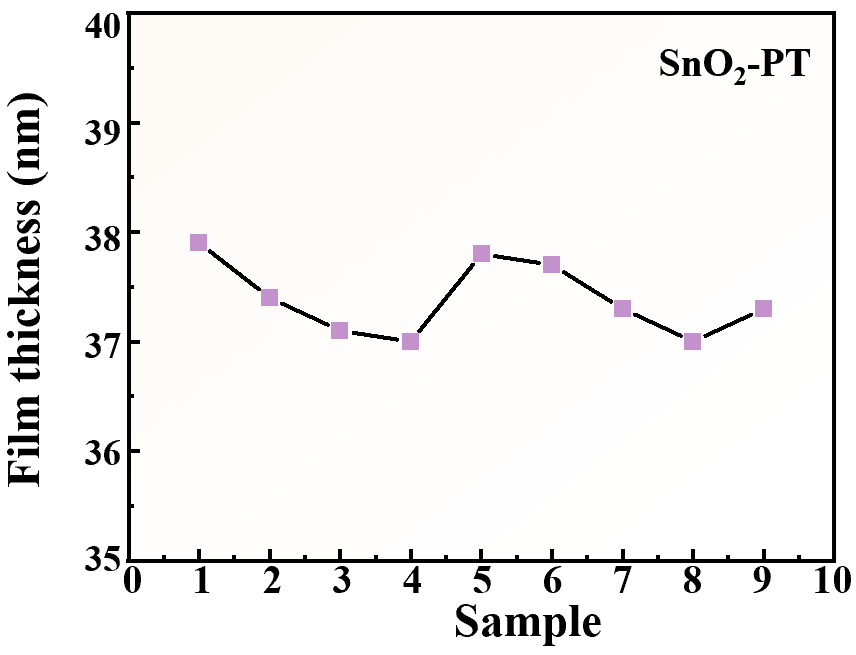


**Figure S7**. The thickness variation curve of SnO_2_-PT films (9 samples).

**
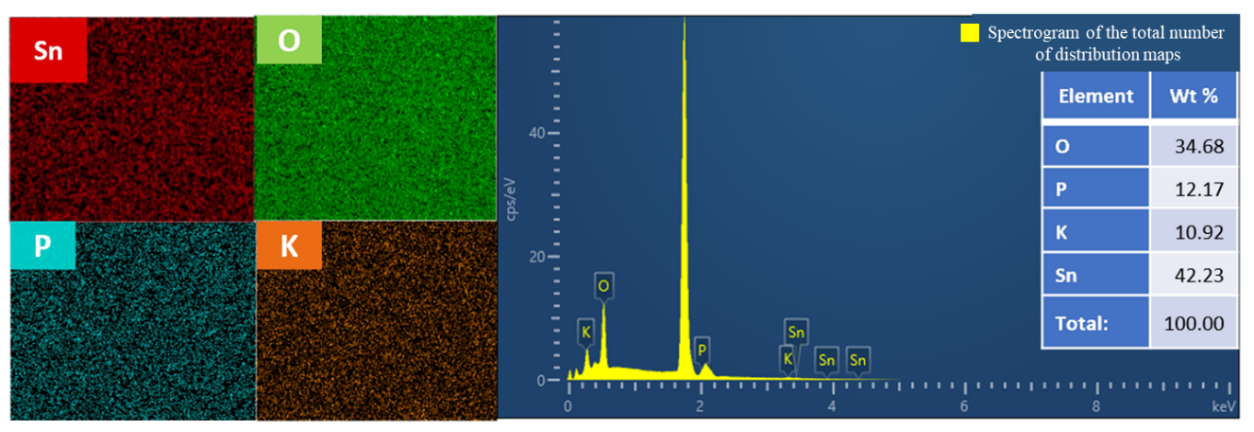
**

**Figure S8**. EDS mapping of Sn, O, P and K elements on the surface of the SnO_2_-PT film.

**
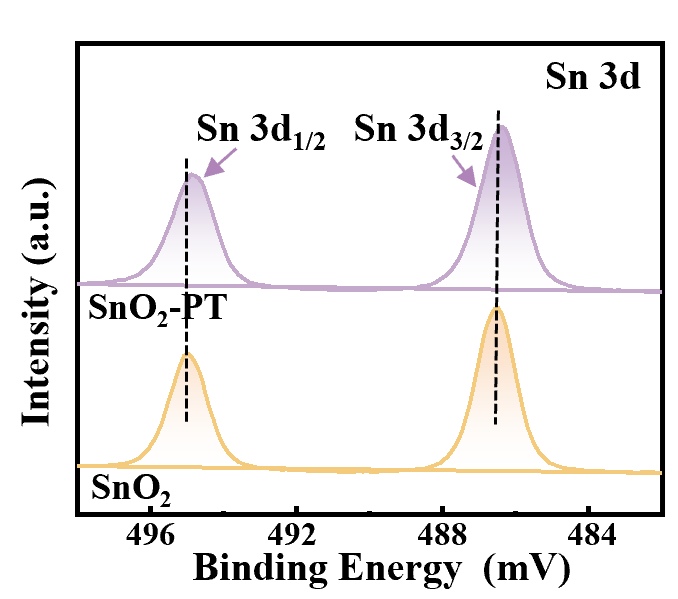
**

**Figure S9**. Sn 3d XPS spectra of SnO_2_ and SnO_2_-PT films.

**
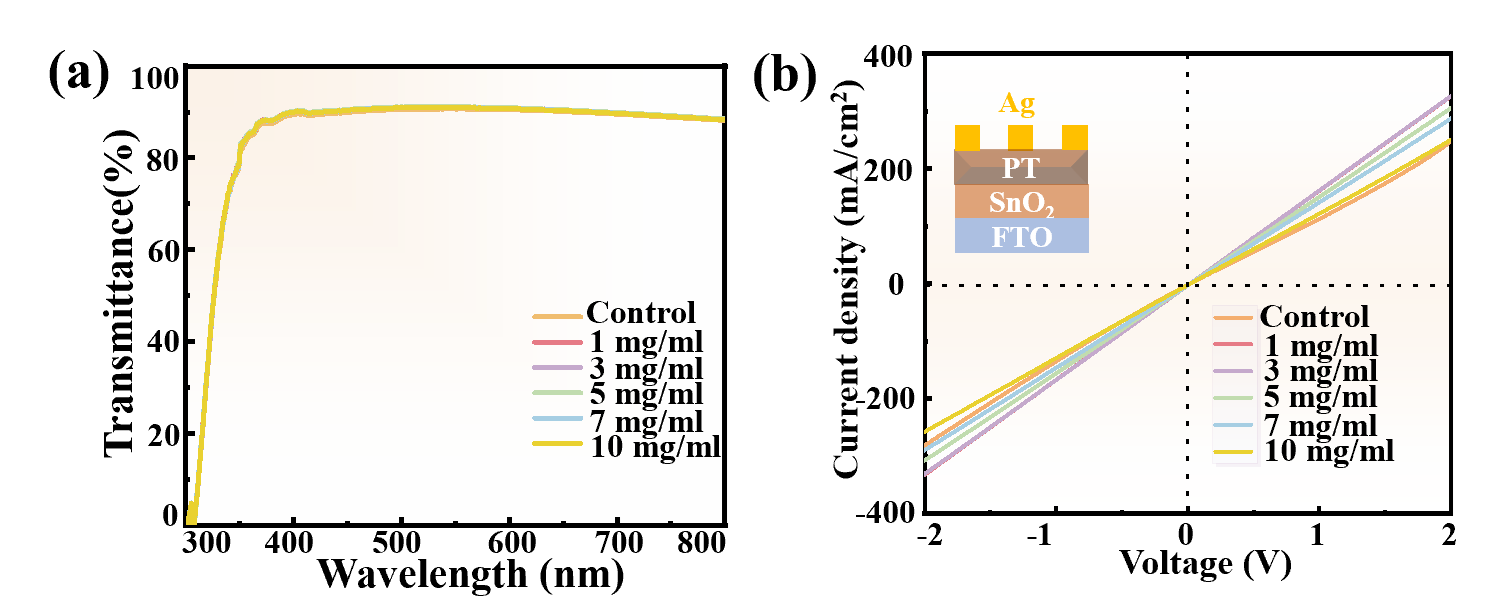
**

**Figure S10**. (a) The transmittance and (b) I-V curves of SnO_2_-PT films modified with different concentrations of PT.


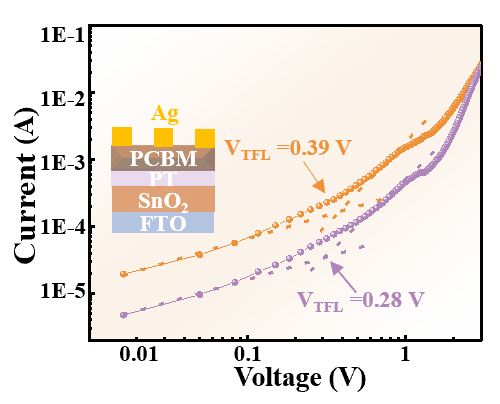


**Figure S11**. The SCLC of SnO_2_ and SnO_2_-PT films.


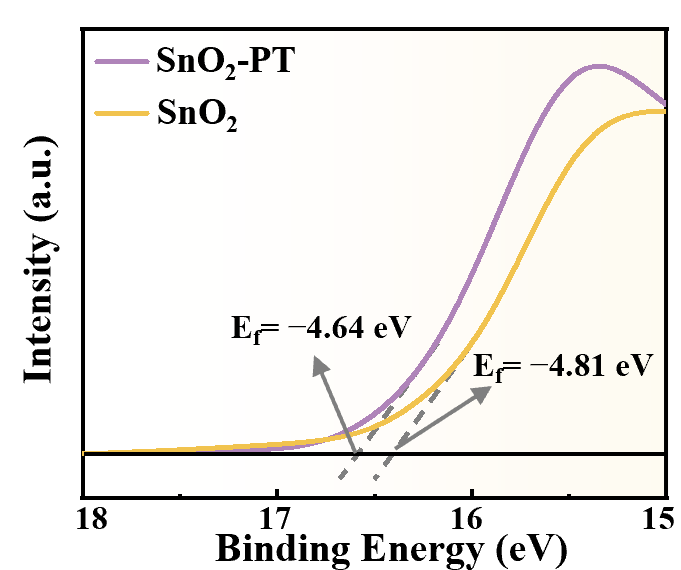


**Figure S12**. The *E_f_* of SnO_2_ and SnO_2_-PT films obtained by secondary-electron cut-off region of UPS spectra.


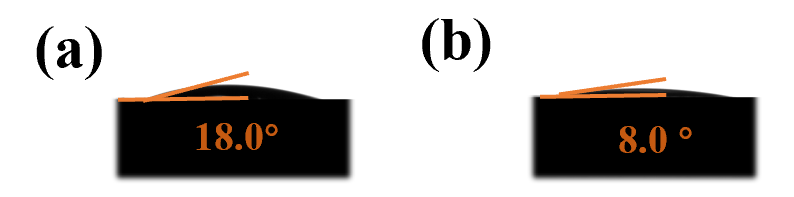


**Figure S13**. Hydrophobic angle of SnO_2_ and SnO_2_-PT films.

**
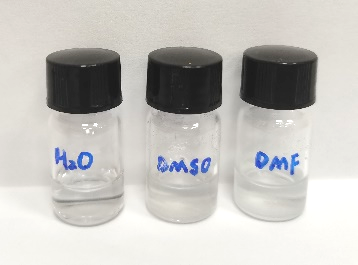
**

**Figure S14**. PT solubility in different solvents (The solution after PT dissolution is clear).


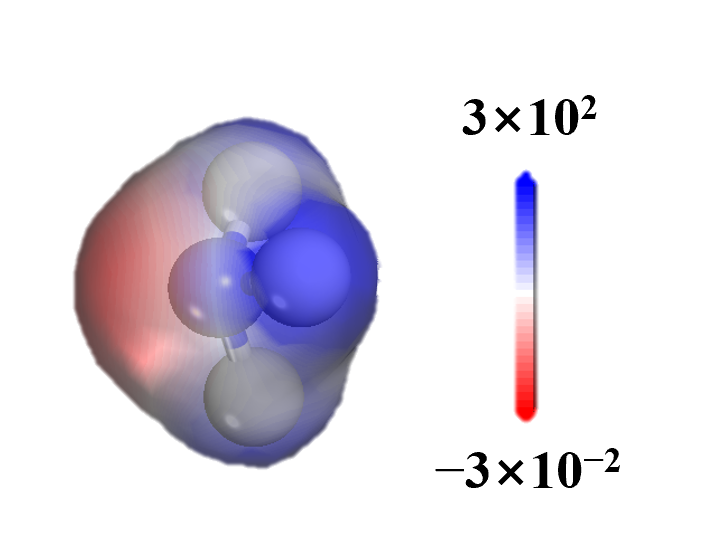


**Figure S15**. The electrostatic surface potential (ESP) map of NH_3_.


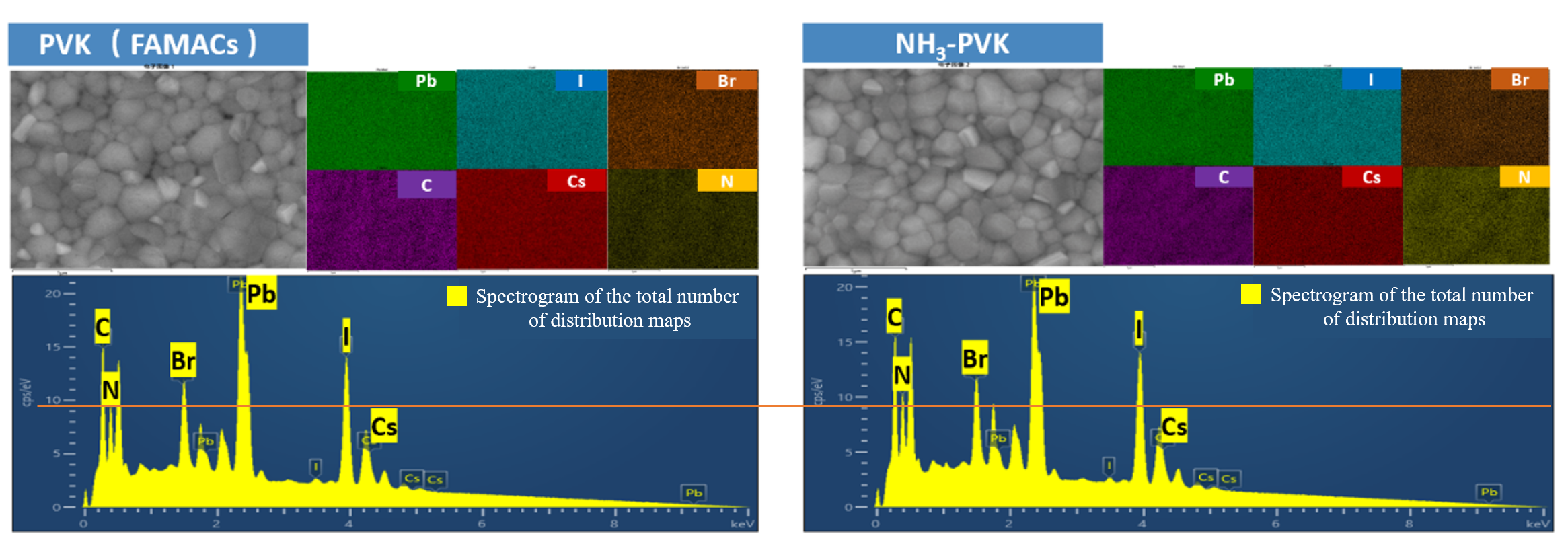


**Figure S16**. EDS mapping images and component distribution spectrogram of PVK and PVK-NH_3_ thin films.


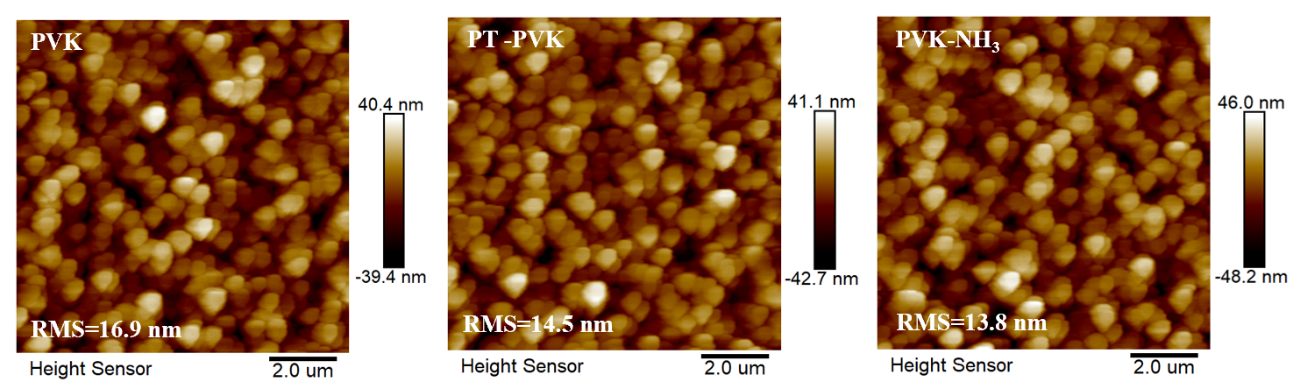


**Figure S17**. AFM images of PVK-NH_3_ thin films.


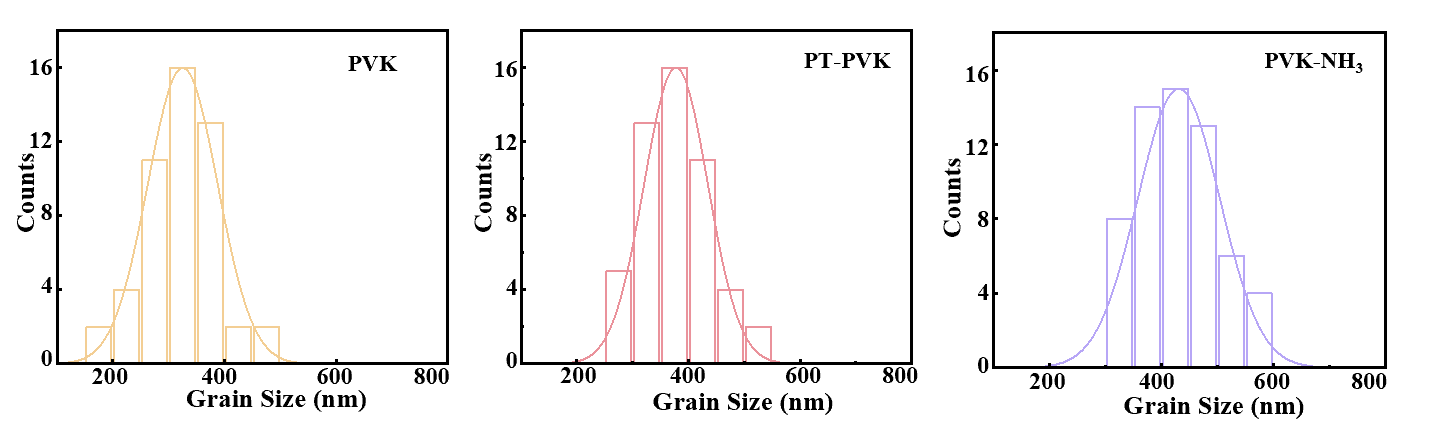


**Figure S18**. The size distribution of PVK, PT-PVK, and PVK-NH_3_ thin films.


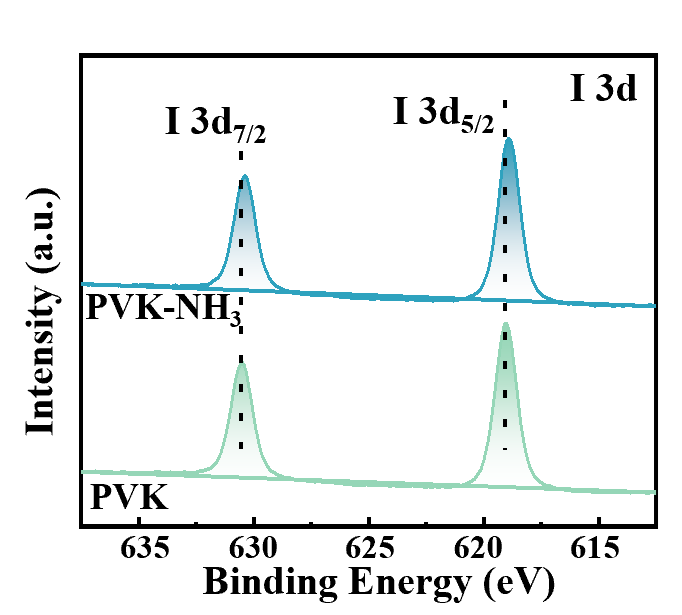


**Figure S19**. I 3d XPS spectra for the PVK and PVK-NH_3_.


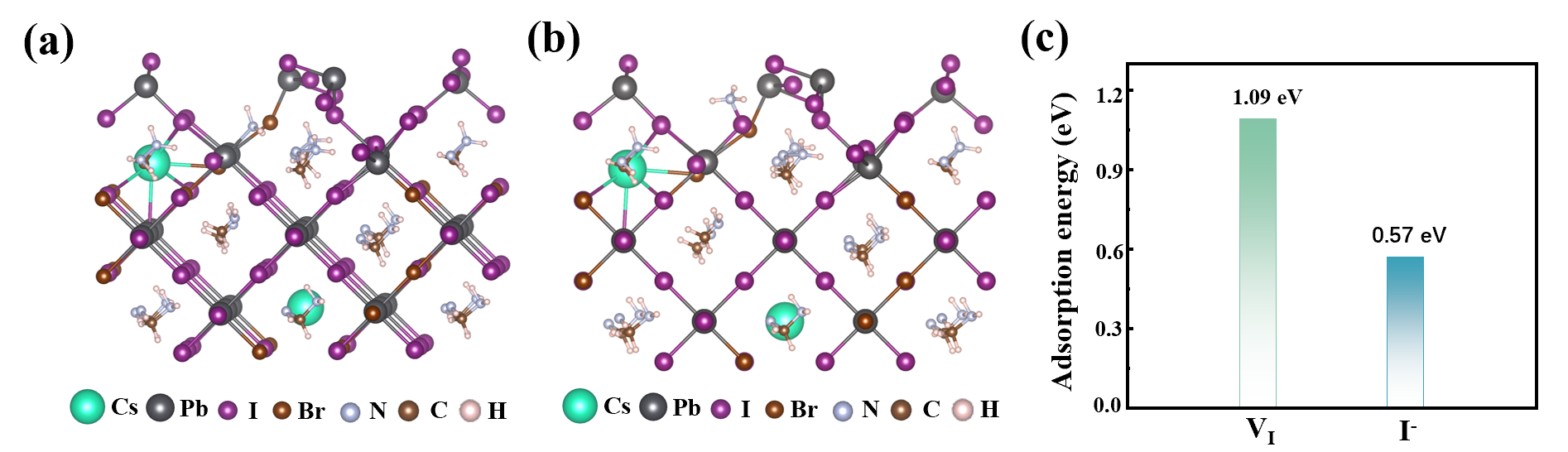


**Figure S20**. (a, b) The adsorption states of NH_3_ on the (001) surface of PVK with V_I_ and I^−^ under DFT simulation. (c)The adsorption energy between NH_3_ and V_I_, I^−^ sites.


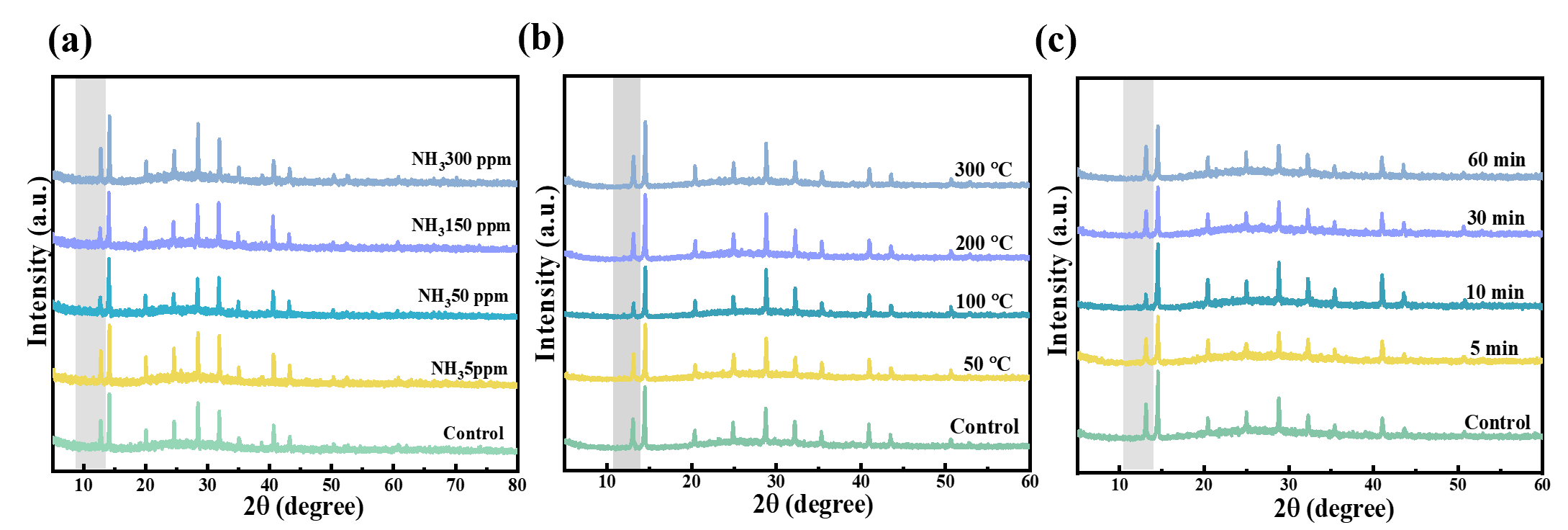


**Figure S21**. (a) XRD patterns of PVK films under different NH_3_ concentrations. (b, c) XRD patterns of PVK films treated at different temperatures and times based on the optimal NH_3_ gas concentration (50 ppm).

**
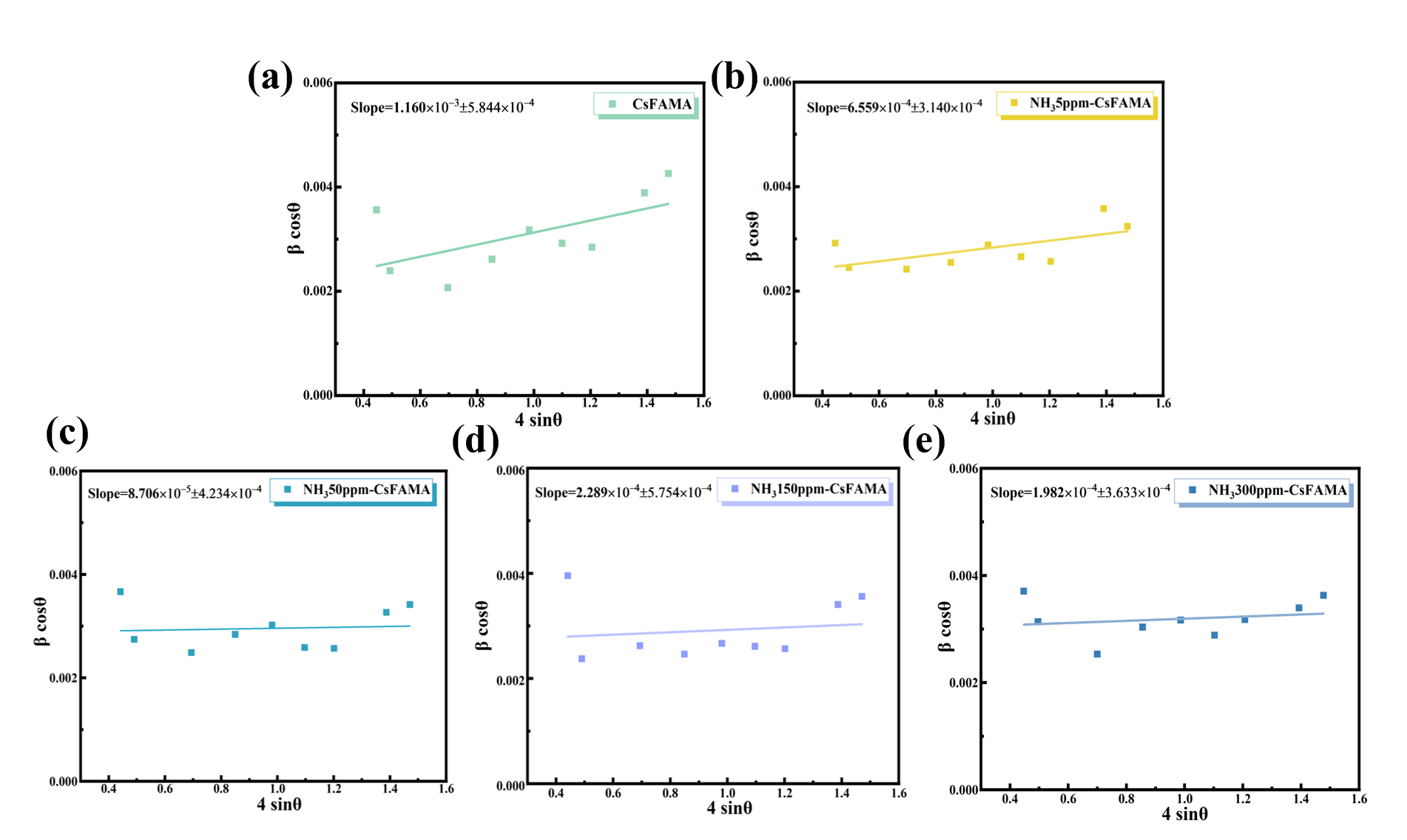
**

**Figure S22**. (a−e) The Williamson-Hall plots of PVK films with different NH_3_ treatment concentrations.


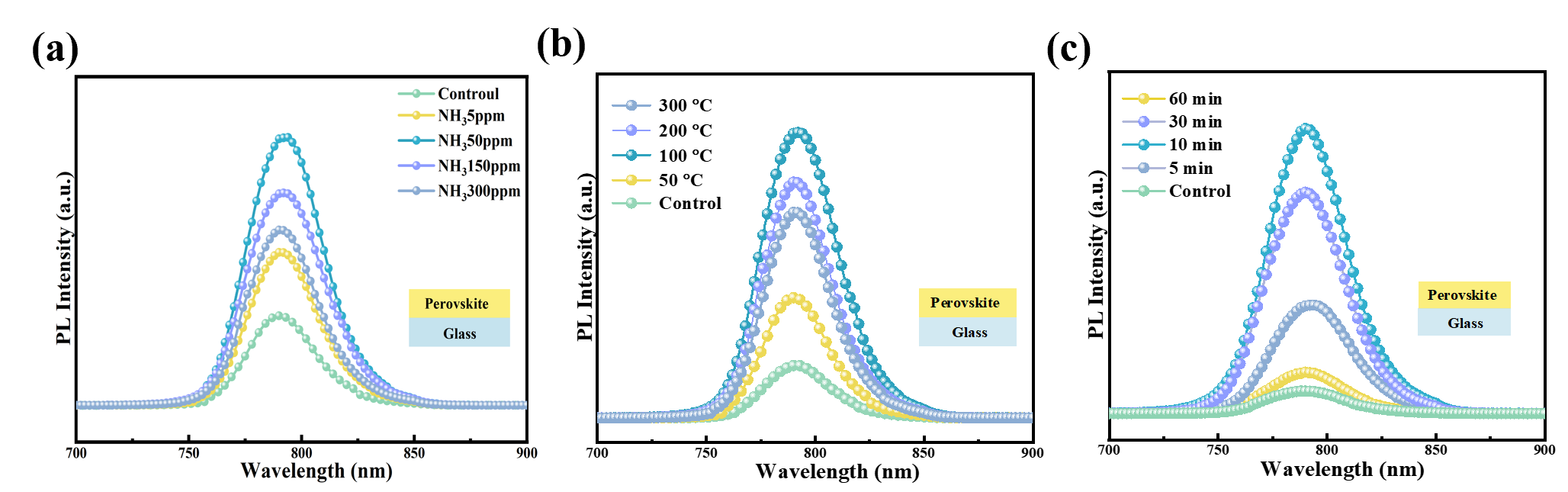


**Figure S23**. (a) PL spectra of PVK films treated with different concentrations of NH_3_. (b, c) Exploring the PL spectra of PVK films treated at different temperatures and times based on the optimal concentration of NH_3_ (50 ppm).


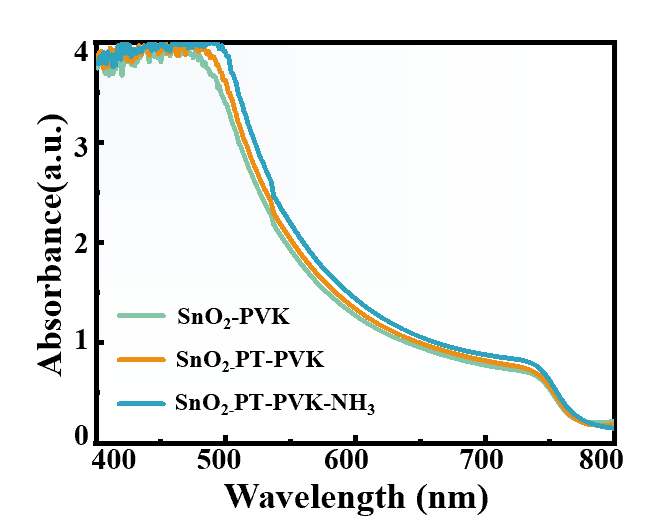


**Figure S24**. UV absorption spectra of Control, PT modification, and dual modification of PT and NH_3_ membranes films.


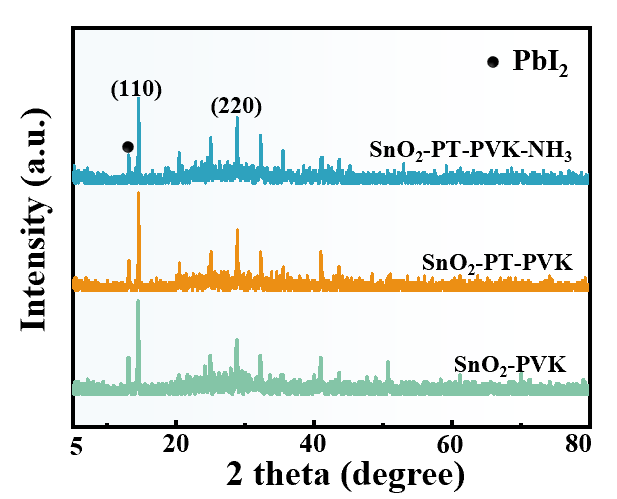


**Figure S25**. XRD patterns of Control, PT modification, and dual modification with PT and NH_3_ films.


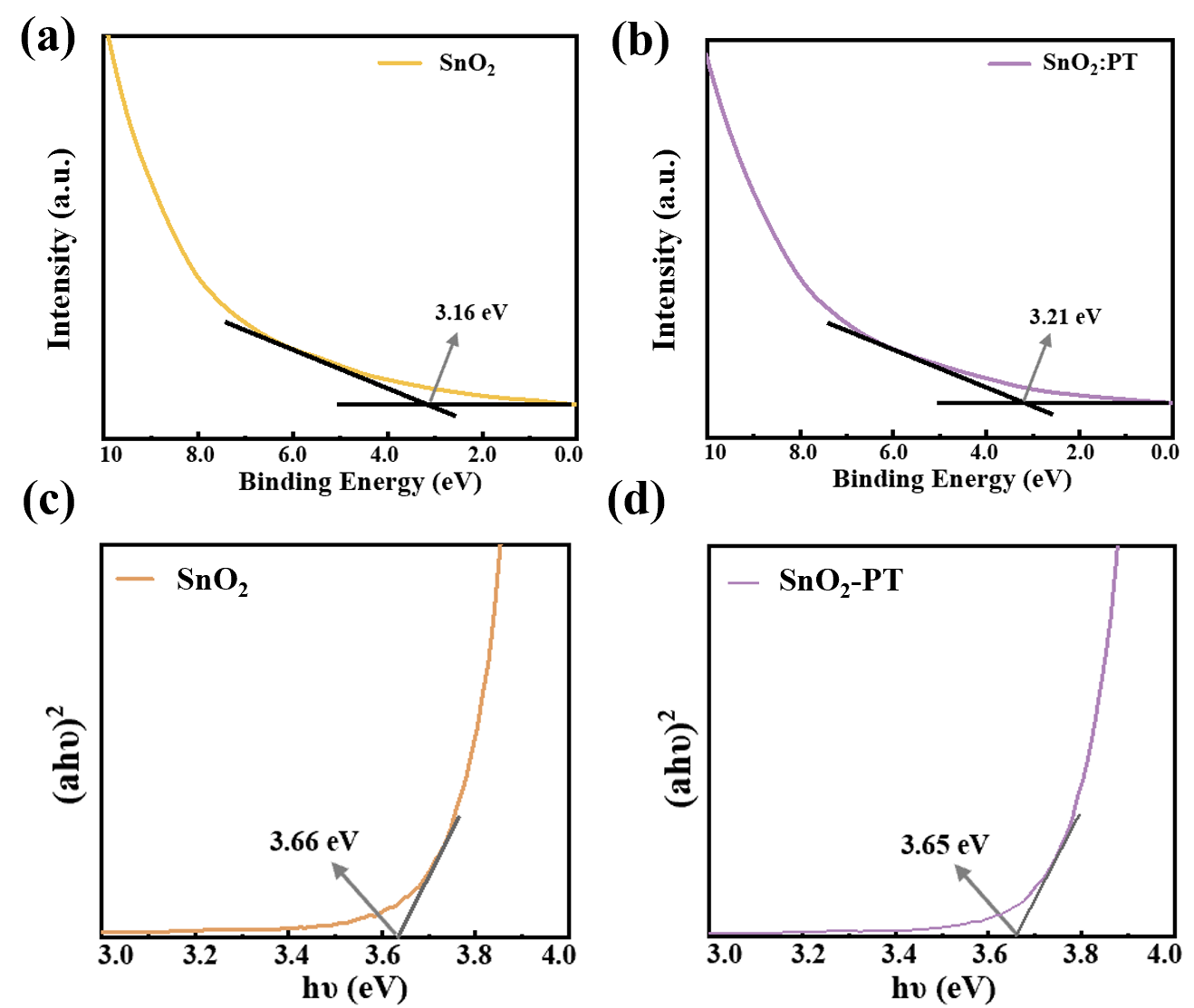


**Figure S26**. (a, b) The (*ahv*)^2^ versus *hv* curves and (c, d) VBM of UPS spectra for SnO_2_ and SnO_2_-PT film.


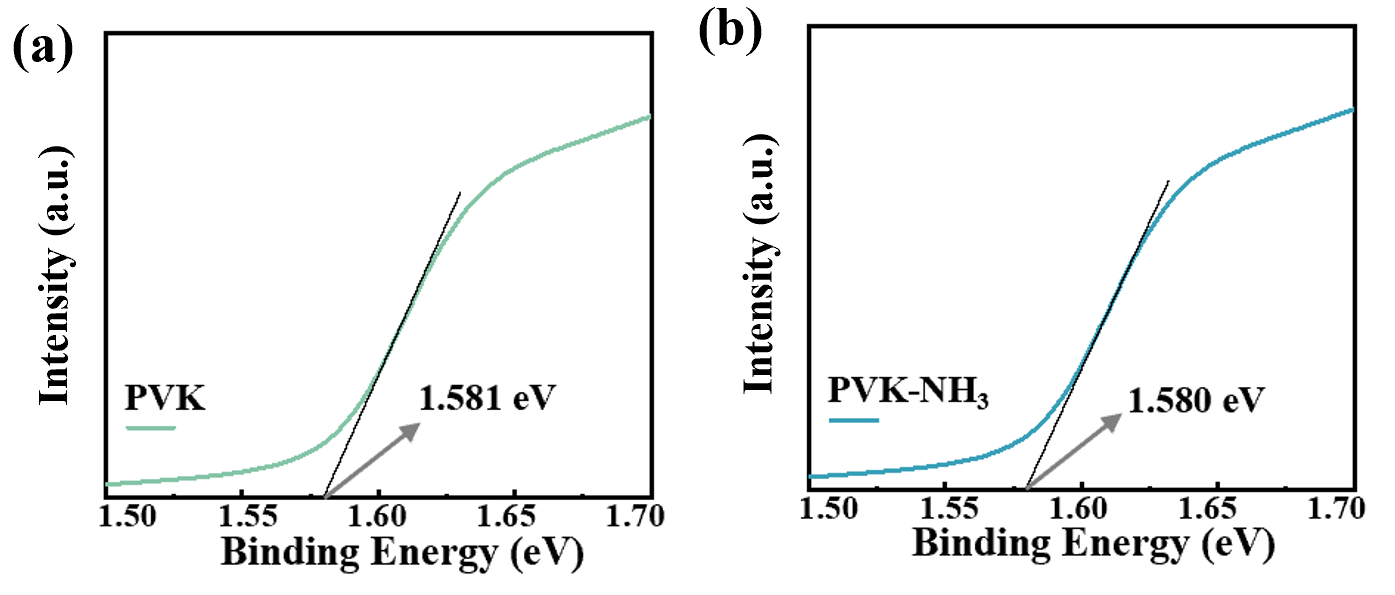


**Figure S27**. (a, b) The (*ahv*)^2^ versus *hv* curve of PVK and PVK-NH_3_ films.


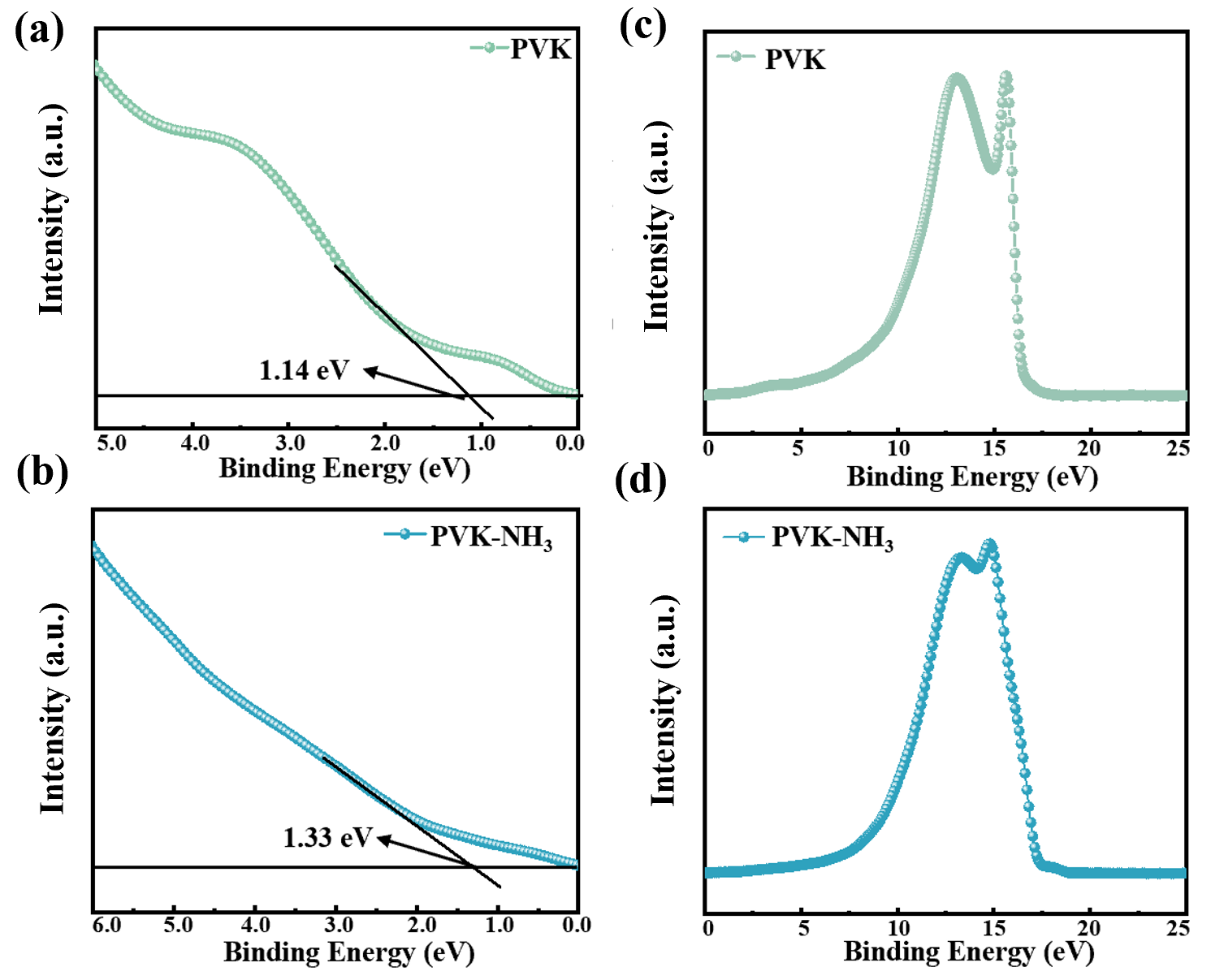


**Figure S28**. (a−d) The VBM and full spectrums of UPS spectra for PVK and PVK-NH_3_ films.


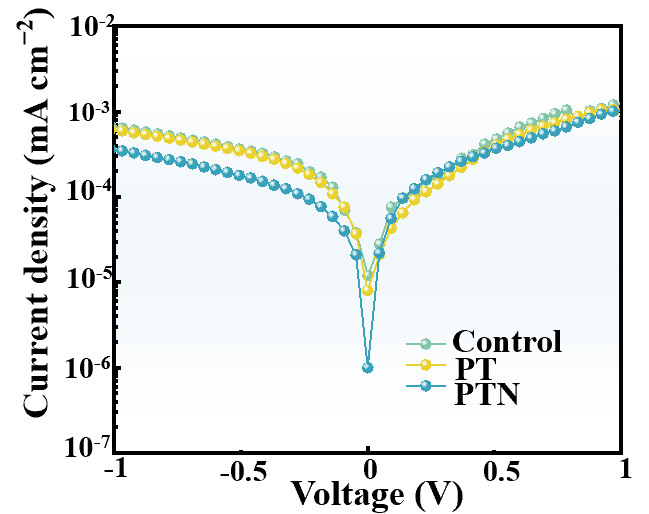


**Figure S29**. Dark J-V curves of the corresponding PSCs.


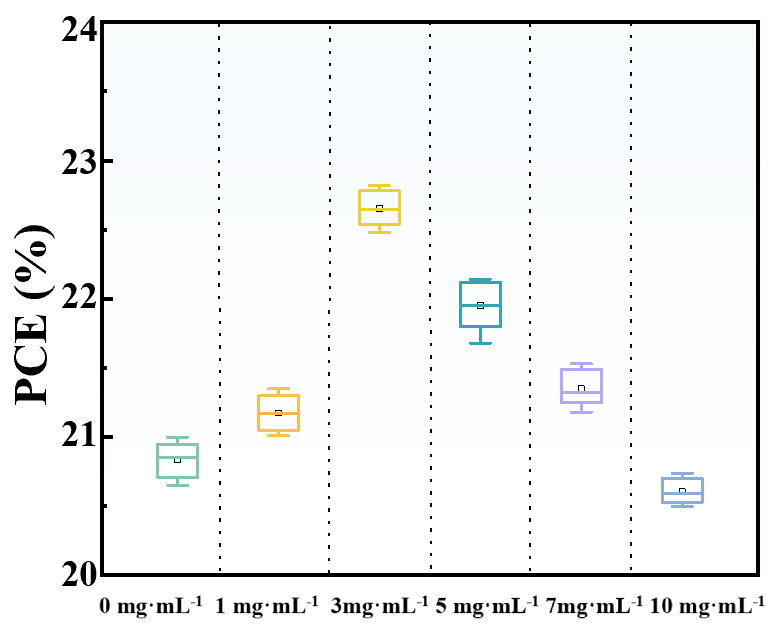


**Figure S30**. Statistics of PCE for the different concentrations of PT surface-treated SnO_2_ of 20 devices, separately.


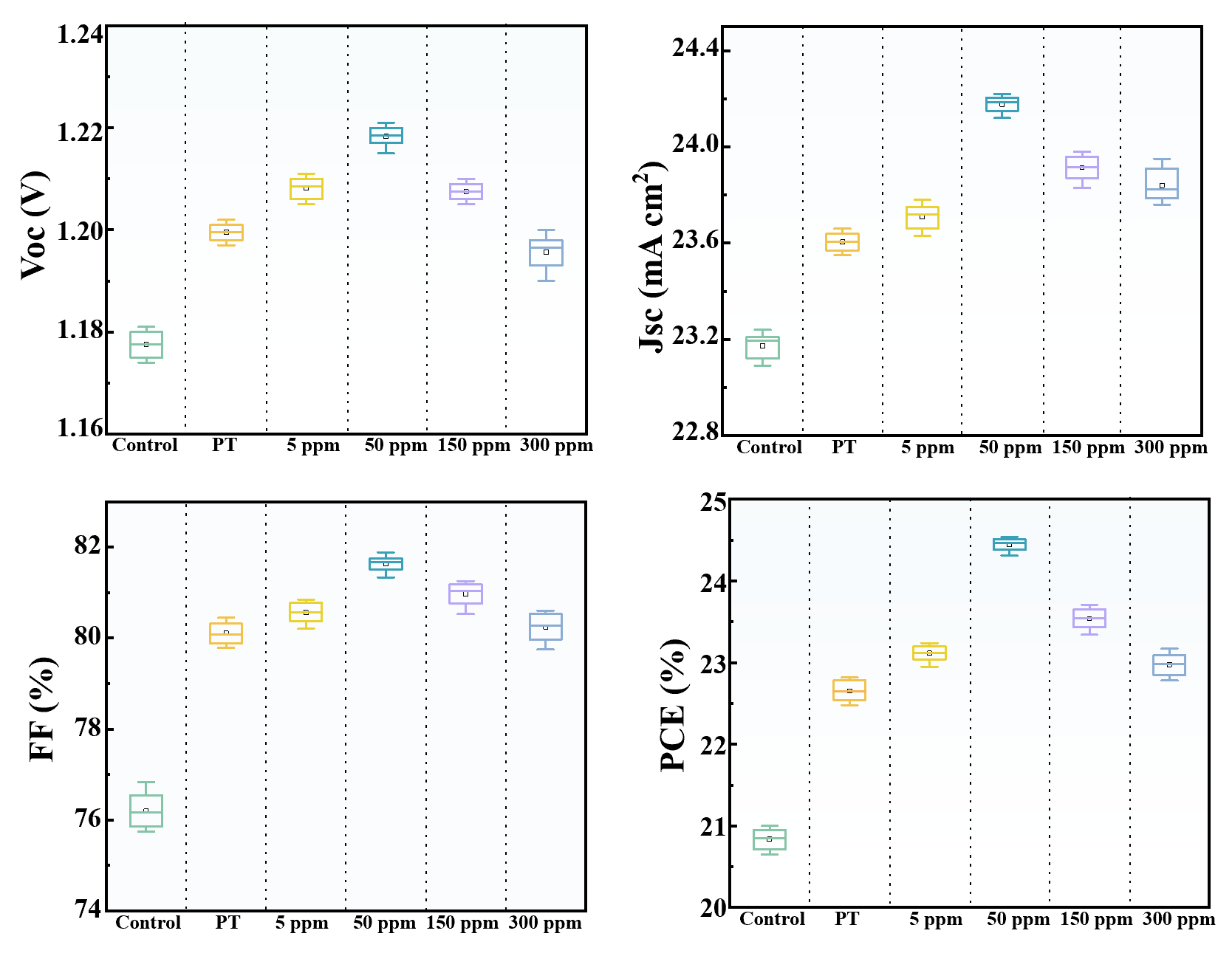


**Figure S31**. Statistics of V_OC_, J_SC_, FF and PCE for Control, PT, and different gas concentrations of 20 devices.


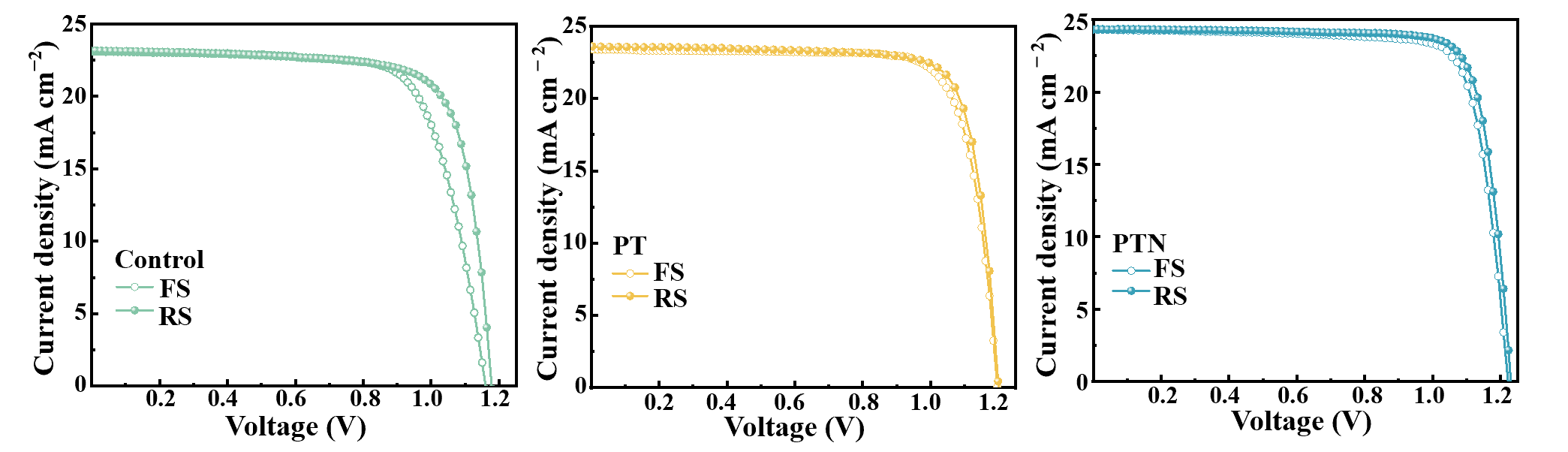


**Figure S32**. The forward and reverse scan J-V curves of Control, PT, and PTN PSCs.


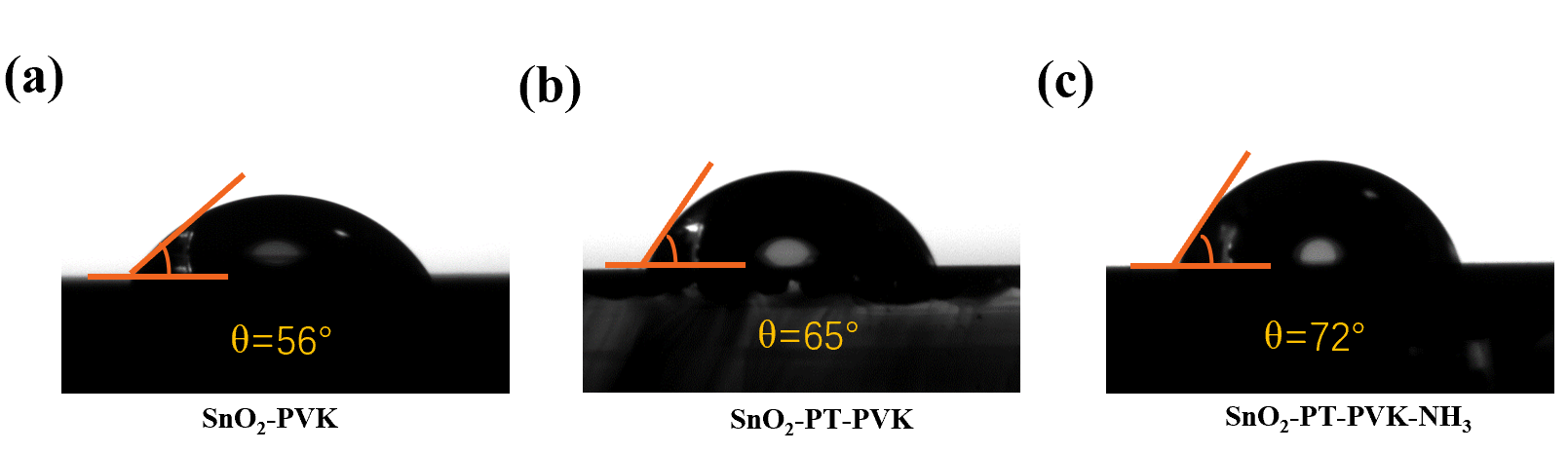


**Figure S33**. (a−c) Hydrophobic angle images of Control, PT modification, and dual modification of PT and NH_3_ membranes.


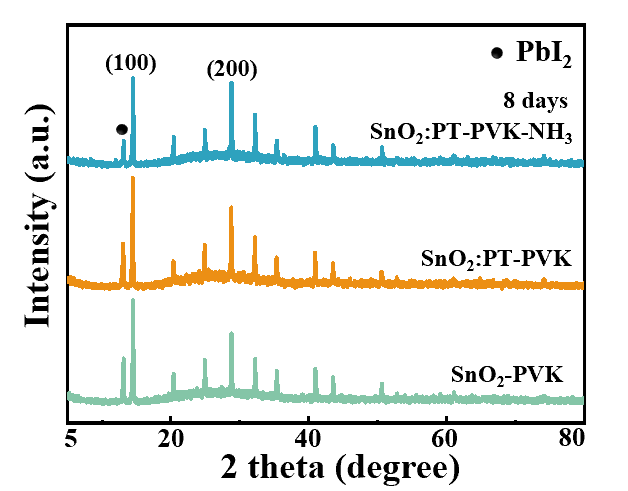


**Figure S34**. Aging (80 days) XRD patterns of Control, PT, and PTN films without encapsulation under air conditions.

**Table S1** The average grain size of the pristine SnO_2_ (SnO_2_1-4) and SnO_2_-PT films (SnO_2_-PT1-4).

| Sample | Average grain size (nm) |
| --- | --- |
| SnO_2_-1 | 22.34 |
| SnO_2_-2 | 22.92 |
| SnO_2_-3 | 22.80 |
| SnO_2_-4 | 21.76 |
| SnO_2_-PT 1 | 22.81 |
| SnO_2_-PT 2 | 22.35 |
| SnO_2_-PT 3 | 22.55 |
| SnO_2_-PT 4 | 21.80 |

**Table S2** Electrical conductivity of SnO_2_ films treated with different PT concentrations.

| PT concentration | $\boldsymbol{\sigma}$ (S·cm^−1^) |
| --- | --- |
| 0 mg·mL^−1^ | 3.7×10^−6^ |
| 1 mg·mL^−1^ | 4.89×10^−6^ |
| 3 mg·mL^−1^ | 4.93×10^−6^ |
| 5 mg·mL^−1^ | 4.59×10^−6^ |
| 7 mg·mL^−1^ | 4.32×10^−6^ |
| 10 mg·mL^−1^ | 3.82×10^−6^ |

Based on I-V testing, the conductivity of SnO_2_ thin films treated with different concentrations of PT was calculated by $\sigma=\frac{d}{AR}$, where d is the thickness of the film (30 nm), A is the effective area of the device (0.1 cm^2^), and R is the resistance calculated by V=IR.

**Table S3** The lifetime parameters fitted from fitting curves of the TRPL measurements (SnO_2_-PVK and SnO_2_-PT-PVK).

| Samples | A_1_ (%) | τ_1_ (ns) | A_2_ (%) | τ_2_ (ns) | Weighted τ_ave_ (ns) |
| --- | --- | --- | --- | --- | --- |
| SnO_2_-PVK | 18.94 | 12.02 | 81.06 | 107.02 | 104.59 |
| SnO_2_-PT-PVK | 21.31 | 8.23 | 78.69 | 77.18 | 75.24 |

The TRPL curves are fitted by the bi-exponential decay function I (τ)=A_1_exp(-t/τ_1_) + A_2_exp(-t/τ_2_), where A_1_ and A_2_ are the relative decay amplitudes, and τ_1_ and τ_2_ correspond to the fast and slow decay lifetimes, respectively.

**Table S4** Element content of PVK and PVK-NH_3_ thin films.

| Samples | C(Wt%) | N (Wt%) | Br (Wt%) | I (Wt%) | Cs (Wt%) | Pb(Wt%) |
| --- | --- | --- | --- | --- | --- | --- |
| PVK | 9.67 | 3.21 | 5.79 | 49.38 | 2.37 | 29.67 |
| PVK-NH_3_ | 9.70 | 3.76 | 5.71 | 48.81 | 2.39 | 29.63 |

**Table S5** The lifetime parameters fitted from fitting curves of the TRPL measurements (PVK and PVK-NH_3_).

| Samples | A_1_ (%) | τ_1_ (ns) | A_2_ (%) | τ_2_ (ns) | Weighted τ_ave_ (ns) |
| --- | --- | --- | --- | --- | --- |
| PVK (CsFAMA) | 39.80 | 7.36 | 60.20 | 81.03 | 76.86 |
| PVK-NH_3_ | 43.90 | 8.60 | 56.10 | 120.54 | 114.62 |

**Table S6** Summary of efficiency of PSCs treated with vapour/gas.

| Absorption layer | ETL | Gas treatment | PCE (%) | Ref |
| --- | --- | --- | --- | --- |
| MAPbI_3_ | TiO_2_ | methylamine  vapour | 14.90% | [8] |
| Cs_0.05_(MA_0.17_FA_0.83_)_0.95_  Pb(I_0.83_Br_0.17_)_3_ | SnO_2_ | 4-fluoroaniline vapour | 20.48% | [9] |
| Cs_0.05_(FA_0.85_MA_0.15_)_0.95_  Pb(I_0.85_B_r0.15_)_3_ | PCBM | 1H,1H,2H,2H  perfluorodecanethiol vapour | 21.79% | [10] |
| FA_0.98_EDA_0.01_SnI_3_ | C_60_ | ethylenediamine vapour | 11.29% | [11] |
| MAPbI_3_ | C_60_ | ethylenediamine vapour | 20.18% | [12] |
| FAPbI_3_ | SnO_2_ | CS_2_ vapour | 25.20% | [13] |
| Cs_0.05_(MA_0.90_FA_0.05_)_0.95_  Pb(I_0.90_Br_0.05_)_3_ | SnO_2_ | pure NH_3_ gas | **24.51%** | **This work** |

**Table S7** The best-performance and statistic photovoltic parameters of 20 devices prepared under different conditions.

| Samples | V_OC_ (V) | J_SC_ (mA·cm^−2^) | FF (%) | PCE (%) |
| --- | --- | --- | --- | --- |
| Control | 1.179 | 23.22 | 76.63 | 20.98 |
| 0 ppm (PT) | 1.201 | 23.66 | 80.32 | 22.82 |
| 5 ppm | 1.211 | 23.76 | 80.78 | 23.24 |
| 50 ppm | 1.229 | 24.36 | 81.88 | 24.51 |
| 150 ppm | 1.217 | 23.98 | 81.23 | 23.70 |
| 300 ppm | 1.202 | 23.84 | 80.89 | 23.17 |

**Table S8** The best-performance and statistic photovoltic parameters of the Control，PT, and PTN PSCs in FS and RS directions.

| Samples |  | V_OC_ (V) | J_SC_ (mA·cm^−2^) | FF (%) | PCE (%) |
| --- | --- | --- | --- | --- | --- |
| Control  (HI=6.62%) | FS | 1.164 | 23.15 | 72.71 | 19.59 |
|  | RS | 1.179 | 23.22 | 76.63 | 20.98 |
| PT  (HI=3.11%) | FS | 1.198 | 23.40 | 78.88 | 22.11 |
|  | RS | 1.201 | 23.66 | 80.32 | 22.82 |
| PTN  (HI=2.89%) | FS | 1.221 | 24.25 | 80.35 | 23.80 |
|  | RS | 1.229 | 24.36 | 81.88 | 24.51 |

The HI factors of the corresponding PSCs were calculated based on HI=(PCE_reverse_−PCE_forward_)/PCE_reverse_ ×100% and marked in the J-V image.

**Table S9** The initial device performance parameters of Control, PT and PTN devices with PTAA for the thermal stability test.

| Samples | V_OC_ (V) | J_SC_ (mA·cm^−2^) | FF (%) | PCE (%) |
| --- | --- | --- | --- | --- |
| Control | 1.141 | 22.52 | 67.30 | 17.29 |
| PT | 1.170 | 23.02 | 68.24 | 18.37 |
| PTN | 1.198 | 23.56 | 71.87 | 20.28 |

**Table S10** The ratio of peak intensities of PbI_2_/(100) of corresponding films under different aging conditions.

| PbI_2_/(100) | Control | PT | PTN |
| --- | --- | --- | --- |
| Long term stability | 0.48 | 0.35 | 0.33 |
| Thermal stability | 0.81 | 0.76 | 0.70 |
| Light stability | 1.17 | 0.85 | 0.82 |

**References**

[1] P. Giannozzi, O. Andreussi, T. Brumme, O. Bunau, M. B. Nardelli, M. Calandra, R. Car, C. Cavazzoni, D. Ceresoli, M. Cococcioni, N. Colonna, I. Carnimeo, A. Dal Corso, S. de Gironcoli, P. Delugas, R. A. DiStasio, A. Ferretti, A. Floris, G. Fratesi, G. Fugallo, R. Gebauer, U. Gerstmann, F. Giustino, T. Gorni, J. Jia, M. Kawamura, H. Y. Ko, A. Kokalj, E. Küçükbenli, M. Lazzeri, M. Marsili, N. Marzari, F. Mauri, N. L. Nguyen, H. V. Nguyen, A. Otero-de-la-Roza, L. Paulatto, S. Poncé, D. Rocca, R. Sabatini, B. Santra, M. Schlipf, A. P. Seitsonen, A. Smogunov, I. Timrov, T. Thonhauser, P. Umari, N. Vast, X. Wu, S. Baroni, *J. Phys.-Condes. Matter* **2017**, *29*, 465901.

[2] P. Giannozzi, S. Baroni, N. Bonini, M. Calandra, R. Car, C. Cavazzoni, D. Ceresoli, G. L. Chiarotti, M. Cococcioni, I. Dabo, A. Dal Corso, S. de Gironcoli, S. Fabris, G. Fratesi, R. Gebauer, U. Gerstmann, C. Gougoussis, A. Kokalj, M. Lazzeri, L. Martin-Samos, N. Marzari, F. Mauri, R. Mazzarello, S. Paolini, A. Pasquarello, L. Paulatto, C. Sbraccia, S. Scandolo, G. Sclauzero, A. P. Seitsonen, A. Smogunov, P. Umari, R. M. Wentzcovitch, *J. Phys.-Condes. Matter* **2009**, *21*, 395502.

[3] J. Paier, R. Hirschl, M. Marsman, G. Kresse, *J. Chem. Phys.* **2005**, *122*, 234102.

[4] J. P. Perdew, K. Burke, M. Ernzerhof, *Phys. Rev. Lett.* **1996**, *77*, 3865.

[5] Blochl, *Phys. Rev. B* **1994**, *50*, 17953.

[6] S. Grimme, S. Ehrlich, L. Goerigk, *J. Comput. Chem.* **2011**, *32*, 1456.

[7] S. Grimme, J. Antony, S. Ehrlich, H. Krieg, *J. Chem. Phys.* **2010**, *132*, 154104.

[8] M. J. Zhang, N. Wang, S. P. Pang, L. Lv, C. S. Huang, Z. M. Zhou, F. X. Ji, *ACS Appl. Mater. Interfaces* **2016**, *8*, 31413.

[9] S. H. Zhao, J. S. Xie, G. H. Cheng, Y. R. Xiang, H. Y. Zhu, W. Y. Guo, H. Wang, M. C. Qin, X. H. Lu, J. L. Qu, J. N. Wang, J. B. Xu, K. Y. Yan, *Small* **2018**, *14*, 1803350.

[10] H. Zhang, K. Li, M. Sun, F. L. Wang, H. Wang, A. K. Y. Jen, *Adv. Energy Mater.* **2021**, *11*, 2102281.

[11] Z. Zhang, M. A. Kamarudin, A. K. Baranwal, L. Wang, G. Kapil, S. R. Sahamir, Y. Sanehira, M. M. Chen, Q. Shen, S. Hayase, *ACS Appl. Mater. Interfaces* **2022**, *14*, 36200.

[12] M. I. Haider, H. Hu, T. Seewald, S. Ahmed, M. Sultan, L. Schmidt-Mende, A. Fakharuddin, *Sol. RRL* **2023**, *7*, 2201092.

[13] K. Zhang, Y. Wang, M. Q. Tao, L. T. Guo, Y. R. Yang, J. Y. Shao, Y. Y. Zhang, F. Y. Wang, Y. L. Song, *Adv. Mater.* **2023**, *35*, 2211593.
